# *Arabidopsis* PARC6 Is Critical for Plastid Morphogenesis in Pavement, Trichome, and Guard Cells in Leaf Epidermis

**DOI:** 10.3389/fpls.2019.01665

**Published:** 2020-01-15

**Authors:** Hiroki Ishikawa, Mana Yasuzawa, Nana Koike, Alvin Sanjaya, Shota Moriyama, Aya Nishizawa, Kanae Matsuoka, Shun Sasaki, Yusuke Kazama, Yoriko Hayashi, Tomoko Abe, Makoto T. Fujiwara, Ryuuichi D. Itoh

**Affiliations:** ^1^ Department of Materials and Life Sciences, Faculty of Science and Technology, Sophia University, Tokyo, Japan; ^2^ Nishina Center for Accelerator-Based Science, RIKEN, Wako, Japan; ^3^ Department of Chemistry, Biology and Marine Science, Faculty of Science, University of the Ryukyus, Okinawa, Japan

**Keywords:** chloroplast, leucoplast, plastid development, stromule, stoma

## Abstract

Recently, a recessive *Arabidopsis thaliana* mutant with abundant stromules in leaf epidermal pavement cells was visually screened and isolated. The gene responsible for this mutant phenotype was identified as *PARC6*, a chloroplast division site regulator gene. The mutant allele *parc6-5* carried two point mutations (G62R and W700stop) at the N- and C-terminal ends of the coding sequence, respectively. Here, we further characterized *parc6-5* and other *parc6* mutant alleles, and showed that *PARC6* plays a critical role in plastid morphogenesis in all cell types of the leaf epidermis: pavement cells, trichome cells, and guard cells. Transient expression of PARC6 transit peptide (TP) fused to the green fluorescent protein (GFP) in plant cells showed that the G62R mutation has no or little effect on the TP activity of the PARC6 N-terminal region. Then, plastid morphology was microscopically analyzed in the leaf epidermis of wild-type (WT) and *parc6* mutants (*parc6-1*, *parc6-3*, *parc6-4* and *parc6-5*) with the aid of stroma-targeted fluorescent proteins. In *parc6* pavement cells, plastids often assumed aberrant grape-like morphology, similar to those in severe plastid division mutants, *atminE1,* and *arc6*. In *parc6* trichome cells, plastids exhibited extreme grape-like aggregations, without the production of giant plastids (>6 µm diameter), as a general phenotype. In *parc6* guard cells, plastids exhibited a variety of abnormal phenotypes, including reduced number, enlarged size, and activated stromules, similar to those in *atminE1* and *arc6* guard cells. Nevertheless, unlike *atminE1* and *arc6*, *parc6* exhibited a low number of mini-chloroplasts (< 2 µm diameter) and rarely produced chloroplast-deficient guard cells. Importantly, unlike *parc6*, the chloroplast division site mutant *arc11* exhibited WT-like plastid phenotypes in trichome and guard cells. Finally, observation of *parc6* complementation lines expressing a functional PARC6-GFP protein indicated that PARC6-GFP formed a ring-like structure in both constricting and non-constricting chloroplasts, and that PARC6 dynamically changes its configuration during the process of chloroplast division.

## Introduction

Plastids represent a diverse group of double membrane-bound organelles, with the ability to transdifferentiate, depending on the tissue type and environmental stimuli (Kirk and Tilney-Bassett, 1978; Mullet, 1988; Pyke, 2007; Pyke, 2009). Leaf cells contain a homogeneous population of round to spherical chloroplasts (photosynthetic plastids; 5–10 µm in diameter) that vary in number from several tens to hundreds per cell (López-Juez and Pyke, 2005). Besides these general features, chloroplasts display varied morphology, depending on the cell type and plant species (Kirk and Tilney-Bassett, 1978; Pyke, 2009; Barton et al., 2016). For example, leaf mesophyll cells develop expanded chloroplasts, with a high degree of inner membrane systems. Mesophyll chloroplasts proliferate by binary fission to sufficiently cover the cell surface (Leech and Pyke, 1988). Chloroplasts in leaf bundle sheath cells contain developed thylakoids and granas; in *Arabidopsis thaliana*, leaf bundle sheath chloroplasts are relatively small in size and lower in number than mesophyll chloroplasts (Kinsman and Pyke, 1998; Kandasamy and Meagher, 1999; Tirlapur and König, 2001). In many plant species, leaf pavement cell chloroplasts are structurally underdeveloped and low in density compared with mesophyll chloroplasts (Pyke and Leech, 1994; Barton et al., 2016; Erickson et al., 2017). Furthermore, leaf stomatal guard cells contain small-sized chloroplasts at a high density, with fewer thylakoids but more starch grains, compared with mesophyll cells (Sachs, 1875; Zhao and Sack, 1999; Lawson, 2009).

Leaf epidermis is ideal for studying plastid morphogenesis not only because of the wide variation in chloroplast morphology observed among leaf epidermal cells but also because plastids in leaf epidermal cells can be readily detected by fluorescence microscopy and developmentally tracked in the L1 layer during leaf development. In *Arabidopsis*, three different types of plastids have been reported, including pavement cell chloroplasts, trichome leucoplasts, and guard cell chloroplasts, all of which originate from proplastids in the shoot apical meristem, or more strictly, protodermal plastids with poor thylakoids (early differentiating chloroplasts) in leaf primordia (Pyke and Leech, 1994; Robertson et al., 1995; Barton et al., 2018). The number, shape, distribution, and dynamics of plastids have been relatively well-studied using pavement cell chloroplasts (Pyke and Leech, 1994; Schattat and Klösgen, 2011; Schattat et al., 2011; Schattat et al., 2012; Higa et al., 2014; Fujiwara et al., 2015; Erickson et al., 2017; Fujiwara et al., 2017; Fujiwara et al., 2018), while stomata physiology has long been investigated in guard cell chloroplasts (Lawson, 2009; Taiz et al., 2015). However, fewer studies have been conducted on the morphological aspects of epidermal plastids than on mesophyll chloroplasts. Thus, the regulation of epidermal plastid development and morphology remains largely unknown (Pyke, 1999; Barton et al., 2018).

Leaf epidermal plastids are also suitable for studying stromule biology. Stromules are thin, tubular structures derived from the plastid surface that dynamically extend from or retract back to the plastid bodies at the second level (reviewed in Gray et al., 2001; Kwok and Hanson, 2004; Natesan et al., 2005; Schattat et al., 2015; Hanson and Hines, 2018; Erickson and Schattat, 2018). Stromules are surrounded by double envelope membranes containing the stroma and are effectively visualized using various stroma-targeted fluorescent proteins (e.g., Köhler et al., 1997; Köhler and Hanson, 2000; Haswell and Meyerowitz, 2006; Schattat et al., 2012; Delfosse et al., 2016). Although stromules have been detected in most plastid types, their abundance is higher in plastids of non-photosynthetic tissues such as petals, roots, endosperms, and bulbs or shoot epidermal tissues such as hypocotyl and leaf epidermis (Kwok and Hanson, 2004; Waters et al., 2004; Erickson et al., 2017). Stromule development is responsive to various abiotic and biotic stimuli, such as light, heat, reactive oxygen species, phytohormones, sugars, and pathogens. Stromule biogenesis also involves interactions with the cytoskeleton and other organelles (reviewed in Erickson and Schattat, 2018; Hanson and Hines, 2018). Furthermore, inhibition of chloroplast division causes excessive production of stromules in various tissues, as shown in tomato (*Solanum lycopersicum*) *suffulta* mutant and Arabidopsis *arc3*, *arc5*, *arc6*, *crl*, and *atminE1* mutants (Forth and Pyke, 2006; Holzinger et al., 2008; Chen et al., 2009; Kojo et al., 2009; Fujiwara et al., 2015; Fujiwara et al., 2018). These studies indicate the importance of stromules in plant cells; however, the mechanism of the origin of stromules and their functions in plant cells remains largely unknown (Hanson and Hines, 2018).

Previously, we screened an ethyl methanesulfonate (EMS)-mutagenized population of Arabidopsis FL4-4 plants co-expressing a plastid stroma-targeted cyan fluorescent protein (CFP) and mitochondrial matrix-targeted yellow fluorescent protein (YFP) and isolated two independent recessive mutant lines, *stromule biogenesis altered1* (*suba1*) and *suba2*, with abundant stromules in pavement cells (Itoh et al., 2018). Analysis of *suba2* revealed that the causal gene responsible for the mutant phenotype was *PARC6* (*CDP1*/*ARC6H*), a known chloroplast division-regulator gene (Glynn et al., 2009; Zhang et al., 2009; Ottesen et al., 2010). The PARC6 protein is inserted in the chloroplast inner envelope membrane and functions as a component of the chloroplast division machinery (Zhang et al., 2016; Chen et al., 2018a). The N-terminal region of PARC6 functions as a transit peptide (TP) (Glynn et al., 2009), and the subsequent region is exposed to the stroma, allowing interaction with a chloroplast division site regulator ARC3 (Shimada et al., 2004; Maple et al., 2007; Glynn et al., 2009). The C-terminal region of PARC6 is exposed to the intermembrane space, where it interacts with an outer envelope-localized division protein PDV1 (Miyagishima et al., 2006; Zhang et al., 2016). In *parc6*, the spatial control of FtsZ ring formation (the first event of chloroplast division; Miyagishima et al., 2011) is perturbed, resulting in asymmetric or multiple chloroplast divisions in leaf mesophyll cells (Glynn et al., 2009; Zhang et al., 2009; Ottesen et al., 2010). The *parc6* allele in *suba2*, termed as *parc6-5*, carries two nucleotide substitutions, resulting in G62R and W700stop mutations at the translation level. The G62R mutation is located in the TP of PARC6 (Glynn et al., 2009), whereas the W700stop mutation is present in the C-terminal region. Additionally, mutant analysis indicated that the enlarged size and excessive stromule proliferation phenotypes of *parc6-5* pavement cell plastids are similar to those of other *parc6* alleles, including *parc6-1*, *parc6-3*, and *parc6-4* (Itoh et al., 2018). Our results also indicated that PARC6 interacts with AtMinD1 (also known as ARC11), another chloroplast division site regulator in mesophyll and pavement cells (Marrison et al., 1999; Colletti et al., 2000; Vitha et al., 2003; Fujiwara et al., 2004; Fujiwara et al., 2008; Fujiwara et al., 2009b; Fujiwara et al., 2017). However, unlike *parc6*, *arc11* shows fairly modest pavement cell chloroplast phenotypes (Fujiwara et al., 2017; Itoh et al., 2018).

Isolation of the *parc6-5* (*suba2*) mutant from leaf pavement cells (Itoh et al., 2018) provided us with an opportunity to comprehensively study plastid morphology in the leaf epidermis. Our initial investigation (Itoh et al., 2018) raised several questions. In this paper, we attempted to complement our former study and comprehensively understand PARC6-mediated plastid morphologies in the leaf epidermis. The objectives of this study were three-fold: 1) evaluate the effect of G62R and W700stop mutations in *parc6-5*; 2) conduct a detailed investigation of plastid morphologies during epidermal cell development; and 3) examine the intraplastidic behavior of PARC6.

## Materials and Methods

### Plant Materials and Growth Conditions

*A. thaliana* (L.) Heynh. plants were mainly used in this study to investigate plastid morphologies in leaf epidermal cells. Seeds of plastid division mutants, *parc6-1* (SALK_100009; Glynn et al., 2009; Zhang et al., 2009; Ottesen et al., 2010; generated by Alonso et al., 2003), *parc6-3* (Glynn et al., 2009), *parc6-4* (SALK_138043; Zhang et al., 2009; generated by Alonso et al., 2003), *arc6-3* (CS288; Pyke et al., 1994), and *arc11-1* (CS281; Marrison et al., 1999) were obtained from the Arabidopsis Biological Resource Center (ABRC), Ohio State University, Columbus, OH, USA. Two transgenic Arabidopsis lines [FL4-4 and FL6-4; Columbia (Col) background] expressing organelle-targeted fluorescent proteins as well as offspring derived from crosses between the transgenic lines and mutants (*parc6-1* × FL4-4, *parc6-3* × FL4-4, *parc6-4* × FL4-4, *arc11-1* × FL4-4, and *arc6-3* × FL6-4) were used (Chen et al., 2009; Itoh et al., 2010; Fujiwara et al., 2018; Itoh et al., 2018; see summary in **Table 1**). The *parc6-5* (*suba2*) mutant was isolated from EMS-treated FL4-4 seeds carrying two nucleotide substitutions in the *PARC6* coding sequence, resulting in G62R and W700stop mutations at the protein level (Itoh et al., 2018). The *parc6-1* mutant was crossed with FL6-4 transgenic line in this study. To analyze plastid division mutants, Col, FL4-4, or FL6-4 plants were correspondingly used as the wild type (WT). Seeds were germinated and grown under daily irradiation from 5:00 to 21:00, as described previously (Fujiwara et al., 2009b), unless otherwise specified.

**Table 1:** List of transgenic *Arabidopsis thaliana* lines^1^ used for organelle labeling experiments in this study.

Plant line	Transgene	Organelle labeling confirmed	Reference
FL4-4^2^	CaMV35Sp::*TP_FtsZ1-1_-CFP*::NOSt, CaMV35Sp::*Pre_mtHSP60_-YFP*::NOSt	Plastid-targeted CFP, mitochondria-targeted YFP	Itoh et al. (2010)
FL6-4^2,3^	CaMV35Sp::*TP_FtsZ1-1_-YFP*::NOSt, CaMV35Sp::*NLS_cry2_-CFP*::NOSt	Plastid-targeted YFP	Chen et al. (2009)
*parc6-1* × FL4-4	Identical to FL4-4	Plastid-targeted CFP, mitochondria-targeted YFP	Itoh et al. (2018)
*parc6-1* × FL6-4	Identical to FL6-4	Plastid-targeted YFP	This study
*parc6-3* × FL4-4	Identical to FL4-4	Plastid-targeted CFP, mitochondria-targeted YFP	Itoh et al. (2018)
*parc6-4* × FL4-4	Identical to FL4-4	Plastid-targeted CFP, mitochondria-targeted YFP	Itoh et al. (2018)
*parc6-5* (parent: FL4-4)	Identical to FL4-4	Plastid-targeted CFP, mitochondria-targeted YFP	Itoh et al. (2018)
*arc11-1* × FL4-4	Identical to FL4-4	Plastid-targeted CFP, mitochondria-targeted YFP	Fujiwara et al. (2017)
*arc6-3* × FL6-4	Identical to FL6-4	Plastid-targeted YFP	Fujiwara et al. (2018)

^1^See also Materials and Methods.

^2^Both FL4-4 and FL6-4 were transformed with T-DNAs carrying two expression cassettes in tandem.

^3^FL6-4 showed almost no CFP but strong YFP signals in leaf cells.

Tobacco (*Nicotiana tabacum* L. cv. Samsun NN) and onion (*Allium cepa* L.) plants were employed for particle bombardment experiments (described below). Tobacco seeds were germinated and grown in soil under continuous white light at 25°C. Onion bulbs were purchased from a local supermarket in Tokyo.

### Transient Expression Analysis

To examine the effect of G62R mutation on the function of PARC6 TP (N-terminal 76 amino acids; Glynn et al., 2009), green fluorescent protein (GFP) was used as a reporter (Chiu et al., 1996). The TP-coding sequence was PCR amplified from the total DNA of Col and *parc6-5* plants using sequence-specific primers containing restriction sites (underlined), H6-17 (5′-AGCGTCGACGCAATGCCAGTAGCTTACAC-3′) and H6-18 (5′-GCGCCATGGCGACGACATGGATACCACCAC-3′). The PCR product (0.25 kb) was treated with *Sal*I and *Nco*I and cloned into the CaMV35S-sGFP(S65T)-nos vector (Isono et al., 1997; provided by Dr. Yasuo Niwa, University of Shizuoka, Japan) under the control of the cauliflower mosaic virus *35S* promoter (CaMV35Sp) to generate two expression constructs, p35S-H-TP_wt_-GFP and p35S-H-TP_G62R_-GFP. These constructs, as well as the undigested vector, were introduced into the epidermal cells of tobacco leaves and onion bulbs *via* particle bombardment using 0.4-µm gold particles (InBio Gold, Hurstbridge VIC, Australia) and the Biolistic PDS-1000/He system (Bio-Rad, Hercules, CA, USA). Bombardments were conducted at 1,100-psi He pressure, with 27-inch vacuum of Hg in the chamber and 6-cm distance to the target tissue. After the bombardment, samples were incubated at 23°C for 6 or 24 h and then observed by fluorescence microscopy, as described below.

### Fluorescence Stereomicroscopy and Epifluorescence Microscopy

Basal parts of leaves including petioles were excised from Arabidopsis seedlings using tweezers. Based on previous observations (e.g., Fujiwara et al., 2004; Fujiwara et al., 2008; Fujiwara et al., 2009a; Fujiwara et al., 2009b; Itoh et al., 2010; Fujiwara et al., 2015; Fujiwara et al., 2017; Fujiwara et al., 2018; Itoh et al., 2018), the adaxial surface of the leaf epidermis in the upper petiole region was used to analyze leaf pavement, trichome, and guard cells in Arabidopsis, unless otherwise specified.

Fluorescence stereomicroscopy was performed using Leica MZ10 F fluorescence microscope (Leica Microsystems, Heidelberg, Germany) equipped with a color CCD camera (model DP26; Olympus, Tokyo, Japan). Fluorescence signals were detected through optical filters using the 0.63× objective lens (Leica Microsystems).

Epifluorescence microscopy was performed using inverted microscopes, IX71 and IX73 (Olympus), equipped with a CMOS camera (model ORCA-flash2.8; Hamamatsu Photonics, Hamamatsu, Japan) and a color CCD camera (model DP73; Olympus), respectively. Emission of fluorescence signals was detected through optical filters, FF01-483/32 (Semrock, Rochester, NY, USA) for CFP, BA510-550 (Olympus) or FF01-545/55 (Semrock) for GFP, FF01-545/55 (Semrock) for YFP, and BA610IF or BA575IF (Olympus) for chlorophyll using 60× [numerical aperture (N.A.) 1.20], 40× (N.A. 1.25), and 20× (N.A. 0.75) objective lenses. Bright field images were obtained with DIC optics.

Fluorescence images of CFP, GFP, YFP, and chlorophyll autofluorescence as well as bright field images were processed using Adobe Photoshop (Adobe Systems, San Jose, CA, USA), as described previously (Fujiwara et al., 2015).

### Measurement of Stomatal Guard Cells and Plastids

Stomatal guard cells were measured as described previously (Fujiwara et al., 2018), except that leaves were sampled from 15:00 to 17:45 in this study. All samples were examined under the same conditions. Depending on the size, chloroplasts in guard cells were categorized as giant chloroplasts, (>6 µm), normal-sized chloroplasts (2–6 µm), and mini-chloroplasts (<2 µm). All chloroplast counting and measurement experiments were performed using at least three biological replicates (i.e., independent leaves).

### Transmission Electron Microscopy (TEM)

TEM was performed by Tokai Electron Microscopy Inc. (Nagoya, Japan), as described previously (Itoh et al., 2018). Briefly, primary leaves of 10-day-old Col, FL4-4, *parc6-1*, and *parc6-5*/*suba2* seedlings were sampled from 10:30 to 12:00 and fixed in 2% paraformaldehyde and 2% formaldehyde in 0.05 M cacodylate buffer (pH 7.4) at 4°C. The fixed leaf samples were washed with 0.05 M cacodylate buffer, postfixed with 2% osmium tetroxide in 0.05 M cacodylate buffer, and dehydrated in a graded ethanol series (50, 70, 90, and 100%). Samples were then embedded in a 70:30 mixture of propylene oxide and Quetol-651 resin (Nisshin EM, Tokyo, Japan). Ultrathin (80 nm thick) sections were prepared using a diamond knife and then stained with 2% uranyl acetate and lead staining solution (Sigma-Aldrich, Tokyo, Japan). Grids were observed using a JEM-1400Plus electron microscope (JEOL, Tokyo, Japan) equipped with a CCD camera (model EM-14830RUBY2, JEOL).

### Complementation of *parc6* Mutant Phenotype With *PARC6-GFP*

A multiple cloning site of pT7Blue (Novagen, Merck-Millipore, Burlington, MA, USA) was ligated into the *Hin*dIII and *Sac*I sites of the pSMAB704 vector (Igasaki et al., 2002; provided Dr. Hiroaki Ichikawa, NIAS, Japan) by simultaneously removing the vector-derived CaMV35Sp and *uidA* gene to yield pSMAB704-T7. A 1.0 kb fragment of the CaMV35S-sGFP(S65T)-nos vector (Isono et al., 1997; provided by Dr. Yasuo Niwa), comprising the full-length *sGFP*(*S65T*) gene and *nos* terminator (NOSt), was ligated to the *Xba*I and *Eco*RI sites of pSMAB704-T7 by simultaneously removing the vector-derived NOSt to yield pSMAB704-T7-GFP. A 3.4-kb DNA fragment comprising 1.0 kb sequence upstream of *PARC6* and the complete coding sequence of *PARC6* was PCR amplified from the pSMAB704-T7-H vector (Itoh et al., 2018) using sequence-specific primers containing restriction sites (underlined), H6-12 (5′-AGTCTAGACGAGCTGCGCGAAGCTAAAC-3′) and H6-8 (5′-GATCTAGACTTCTGTATTTGAATATCGCTTTG-3′). The PCR product was cloned into the pSMAB704-T7-GFP vector at the *Xba*I site using *Escherichia coli* HST04 strain as a host. The resulting binary vector pSMAB-T7-H-GFP was transformed into *Agrobacterium tumefaciens* C58 strain using the freeze-thaw method. An *Agrobacterium* transformant was employed for T-DNA-mediated nuclear transformation of Arabidopsis *parc6-1* (Glynn et al., 2009) and *parc6-4* (Zhang et al., 2009; Itoh et al., 2018) mutants using the floral dip method (Clough and Bent, 1998). A total of 12 transformed seedlings were selected on Murashige and Skoog (MS) medium containing bialaphos (10 µg/mL) and carbenicillin (100 µg/mL). Transgenic plants in the T_1_, T_2_, and T_3_ generations were characterized by epifluorescence microscopy, as described above.

## Results

### Effect of the G62R Mutation on the Subcellular Localization of PARC6

We investigated which of the two mutations (G62R and W700stop) in the N- and C-terminal coding sequence of *parc6-5* mutant allele in the recessive Arabidopsis *suba2* mutant (Itoh et al., 2018) were responsible for excessive stromule formation (*suba* phenotype) in pavement cells of mature leaves. Although it has been previously shown that Gly62 is located within the experimentally confirmed TP region of PARC6 (Glynn et al., 2009), *in silico* analysis of *parc6-5* mutant allele with several protein localization predictors unanimously predicted that PARC6^G62R^ would be located inside the chloroplasts, similar to the WT PARC6 (Itoh et al., 2018). However, experimental evidence to support this finding was lacking. Moreover, prediction programs are not designed to predict a reduction in the chloroplast protein import efficiency (and the resulting accumulation of precursor proteins in the cytosol) and its possible dependency on the plastid type.

**Figure 1:**
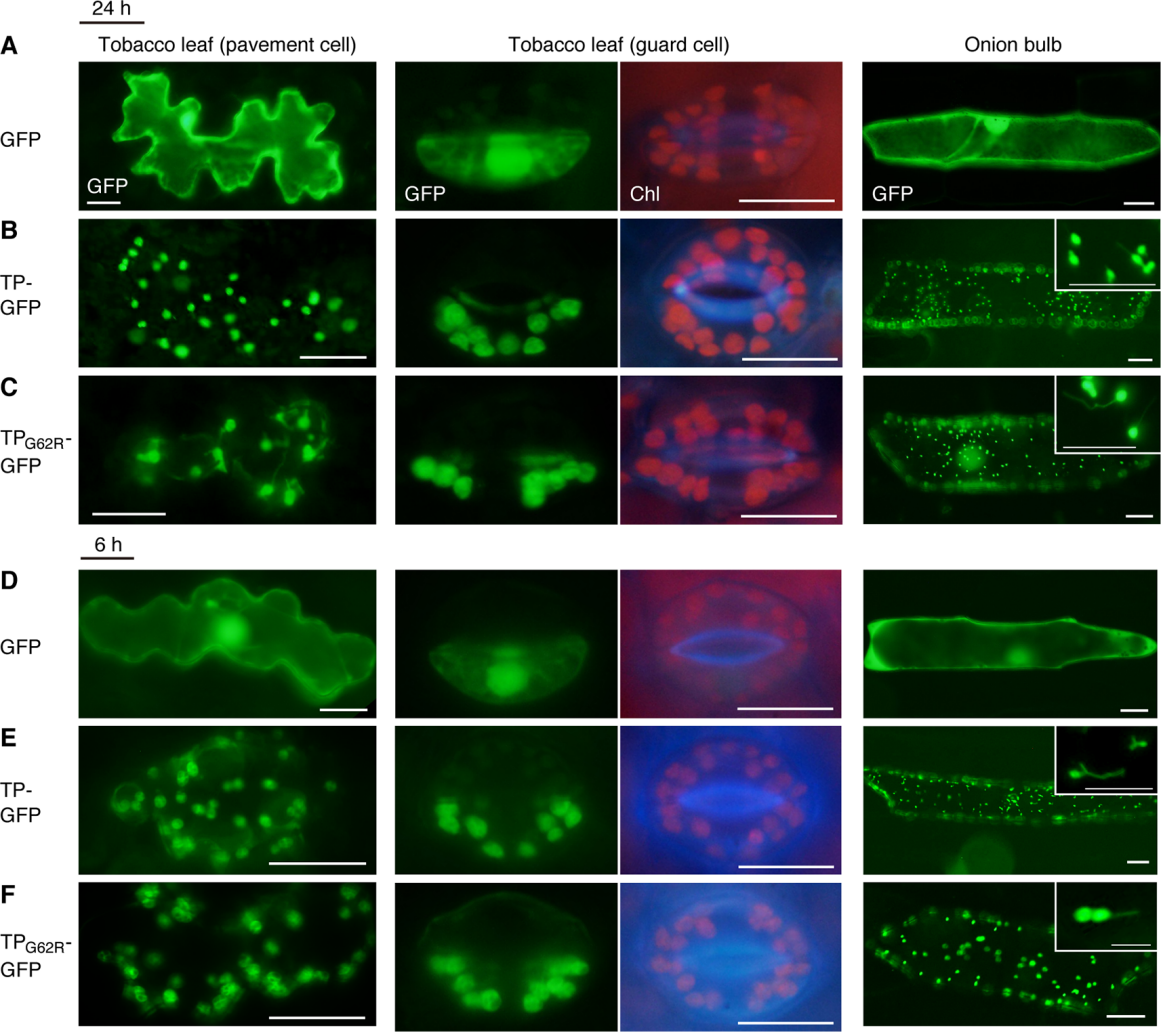
Localization analysis of GFP fused to the mutant transit peptide of PARC6 (TP_G62R_-GFP). GFP with or without a PARC6 transit peptide (TP) sequence from Col or *parc6-5* (TP_G62R_) was transiently expressed in tobacco and onion cells by particle bombardment. **(A**–**F)** Epifluorescence microscopy images of tobacco leaf pavement cells and guard cells and onion epidermal cells at 24 h **(A**–**C)** or 6 h **(D**–**F)** post-bombardment. GFP fluorescence, chlorophyll autofluorescence (Chl), and merged images are shown. Scale bars: 20 µm (all guard cells and insets); 50 µm (others).

To determine the effect of the G62R mutation on the subcellular localization of PARC6, we generated constructs expressing translational fusions of GFP with the mutated PARC6 TP (TP_G62R_-GFP) and its WT counterpart (TP-GFP) or expressing GFP alone (control) under the control of the constitutive CaMV35S promoter. These constructs were introduced into the epidermal cells of tobacco leaves and onion bulbs *via* particle bombardment, and the import of GFP into chloroplasts and leucoplasts, respectively, was monitored. At 24 h post-bombardment, GFP localization was observed by epifluorescence microscopy. The results showed that unfused GFP accumulated in the cytoplasm and nucleoplasm of tobacco pavement cells and guard cells and onion bulb epidermal cells (**Figure 1A**). Fluorescence of TP-GFP fusion protein was detected as particular bodies dispersed throughout the cytoplasm, which overlapped with chlorophyll autofluorescence in tobacco guard cells and thin stromule-like protrusions (distinctive structures of non-green plastids) in onion bulb epidermis (**Figure 1B**). These data support the localization of TP-GFP within the plastid stroma, regardless of the plastid type. Moreover, TP_G62R_-GFP showed the same subcellular localization pattern as the TP-GFP in all cell types (**Figures 1B**, **C**). To investigate the difference in plastid import efficiency between TP-GFP and TP_G62R_-GFP, we further observed GFP localization at 6 h post-bombardment. The localization patterns of the unfused and fused GFPs at 6 h were similar to those of the unfused and fused GFPs, respectively, at 24 h, and no significant difference was detected between the signal intensities (plastid/cytoplasm ratios) of TP-GFP and TP_G62R_-GFP (**Figures 1D**–**F**). Based on these results, we conclude that the G62R substitution in PARC6 encoded by the *parc6-5* mutant allele has no or little effect on the plastid import efficiency of PARC6.

### Plastids in Pavement Cells of *parc6* Mutants

**Figure 2:**
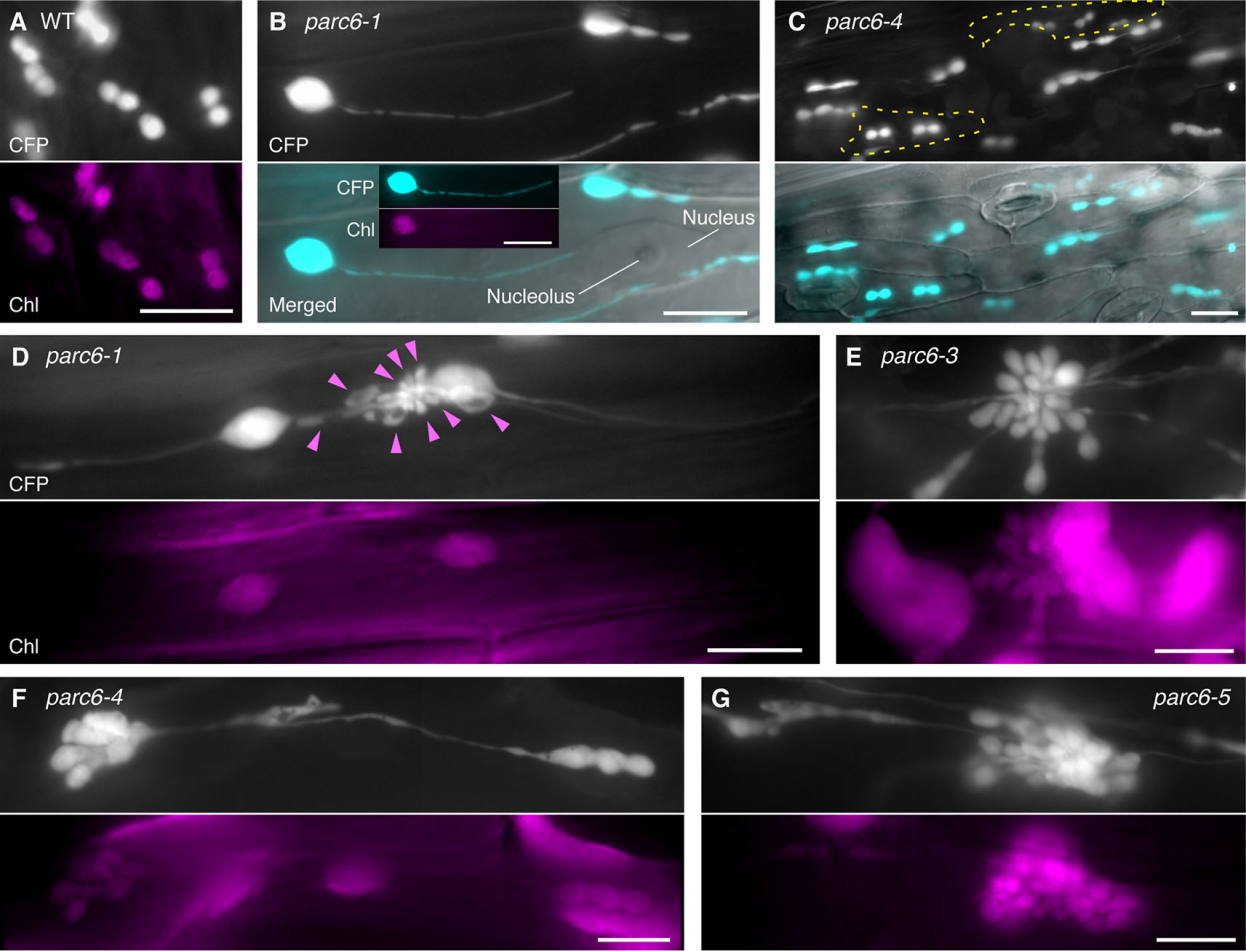
Morphology of plastids in leaf epidermal pavement cells of *parc6* mutants. **(A**–**G)** Images of pavement cells in the 3^rd^ and 4^th^ leaf petioles of 2-week-old wild-type (WT) **(A)**, *parc6-1* **(B**, **D)**, *parc6-3* **(E)**, *parc6-4* **(C**, **F)**, and *parc6-5* **(G)** seedlings. Images of stroma-targeted CFP, chlorophyll autofluorescence, or differential interference contrast (DIC), and merged images of CFP and DIC **(B**, **C)** are shown. Inset in **(B)** indicates chlorophyll autofluorescence in a stromule-producing pavement cell chloroplast. Yellow regions in **(C)** indicate pavement cells with symmetric chloroplast division. Arrowheads in **(D)** indicate chlorophyll-less plastids or bulges. Scale bar = 10 µm.

To complement and complete our previous study (Itoh et al., 2018), we extended our observation of chloroplasts in pavement cells to *parc6* mutants. In the previous study, we used the first and second leaves of 3-week-old seedlings (Itoh et al., 2018), suitable for the analysis of mature pavement cells. In the present study, we used the third and fourth leaves of 2-week-old seedlings, as these are suitable for monitoring the dividing chloroplasts as well as the growing stromules. While stromules in *parc6* pavement cells were excessively elongated compared with those in WT pavement cells, perinuclear stromule attachment, previously reported for WT plants (Erickson et al., 2017; Kumar et al., 2018), was also often detected in *parc6* pavement cells (**Figures 2A**, **B**). Additionally, pavement cell chloroplasts of all *parc6* mutants examined in this study displayed autofluorescence over the entire chloroplast, except the stromule region (see inset in **Figure 2B**), indicating that hyperelongated stromules in *parc6* pavement cells maintained their general properties. Other morphological features of stromules (e.g., branching and preferential elongation along the longitudinal axis of cells) and plastid bodies (e.g., enlargement, heterogeneous size and shape, and multiple constrictions) described previously (Itoh et al., 2018) were also observed in the current study (data not shown). Intriguingly, although at a low frequency, pavement cells exclusively containing relatively normal-sized chloroplasts showed symmetric binary fission (**Figure 2C**; cells surrounded by the yellow dotted line). Although *parc6* mutants are generally recognized as “chloroplast division site” mutants, the present data imply that *parc6* mutants maintain, to some degree, a mechanism for mid-plastid recognition, instead of completely randomly selecting plastid division sites, at least in pavement cells.

All *parc6* mutants showed grape-like clusters of plastids in pavement cells (**Figures 2D**–**G**). Although grape-like clusters showed a widely variable morphology, these clusters were commonly found in *parc6* pavement cells, regardless of the mutant type. Some of the clusters consisted of spherical, ovoid, and amorphous shaped bulges or blobs devoid of chlorophyll (arrowheads in **Figure 2D**); however, other clusters emitted chlorophyll autofluorescence over the whole body, and the majority of chloroplasts in these clusters showed one or more constriction sites each (**Figures 2E**–**G**). These plastids or chloroplasts either developed radially from a single nucleation point or were generated by the accumulation of spherical and ovoid stroma-containing blobs at a single local region. A more careful observation of the latter type revealed that a single cluster included two kinds of blobs, one with and another without chlorophyll autofluorescence, and smaller blobs tended to lack the autofluorescence signal. The morphology of pavement cell chloroplasts in the WT (FL4-4 line; **Figures 2A**, **S1A**) and *arc11* mutant (**Figure S1B**) at the same stage as for *parc6* analysis (**Figures 2B**–**G**) was similar to that observed at the later stage in the WT and *arc11*, respectively, in our previous report (Fujiwara et al., 2017). Chloroplasts in *arc11* pavement cells were seemingly undergoing either symmetric or asymmetric binary fission or multiple fissions (**Figure S1B**); however, no grape-like clusters were detected. In *parc6* pavement cells, we observed both mitochondria and grape-like plastid clusters simultaneously in the same field of view (**Figure S1C**). During the period of observation, blobs in grape-like clusters as well as physically distinct plastids showed motility and shape change. Some mitochondria were stuck and almost immobile in the space between the blobs within the clusters, suggesting occasional attachment between mitochondria and plastid-derived blobs in the clusters.

### Plastids in Trichome Cells of *parc6* Mutants

**Figure 3:**
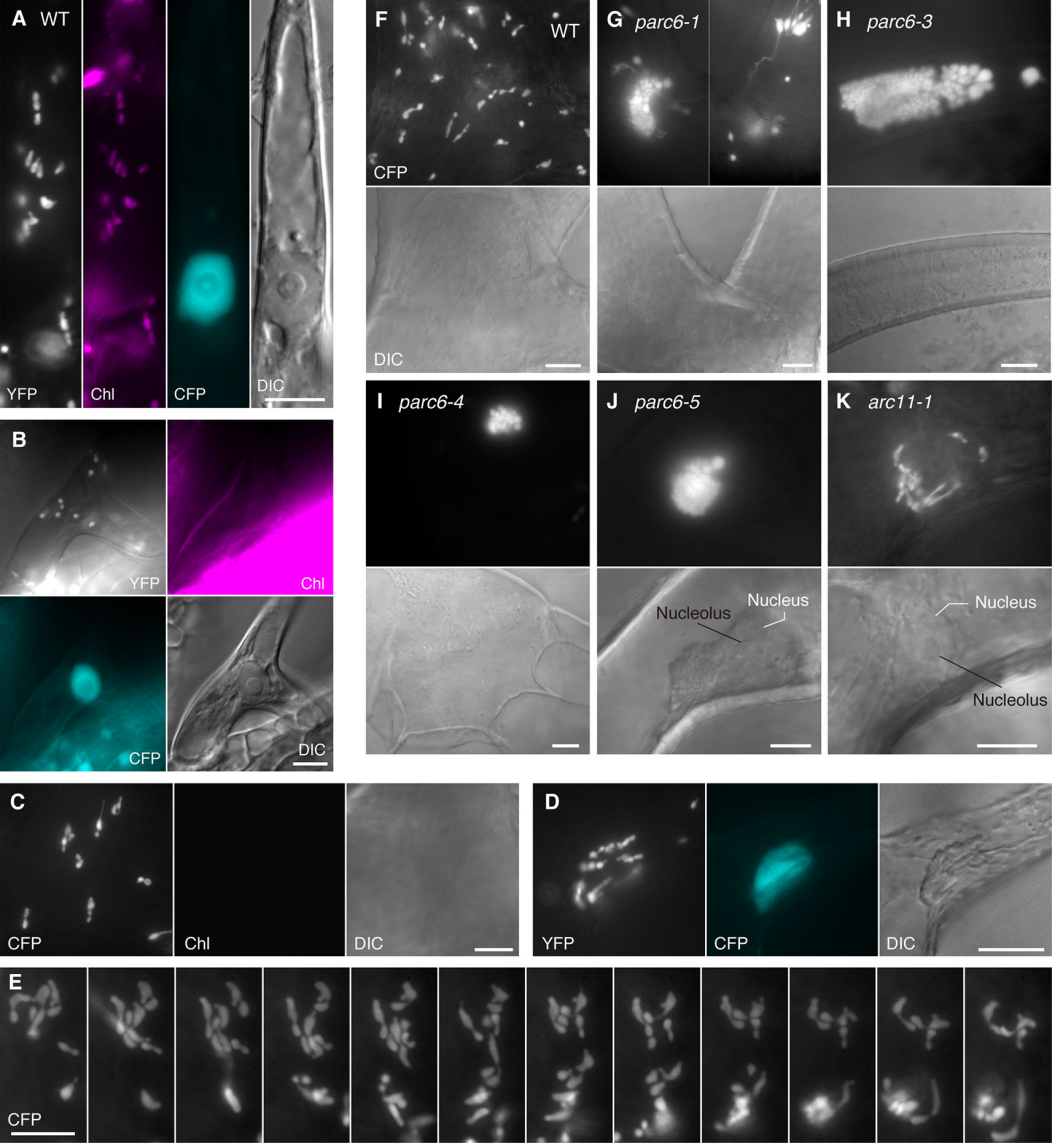
Morphology of plastids in leaf trichome cells of *parc6* and *arc11* mutants. **(A**–**K)** Images of trichomes in leaf petioles of WT **(A**–**F)**, *parc6-1* **(G)**, *parc6-3* **(H)**, *parc6-4* **(I)**, *parc6-5* **(J)**, and *arc11-1* **(K)** seedlings. The 3^rd^ and 4^th^ leaves of 2-week-old seedlings were mainly observed **(A**–**C, F**–**K)**. Images of stroma-targeted CFP or YFP (black-and-white), chlorophyll autofluorescence (magenta) **(A**–**C)**, nucleus-targeted CFP (cyan) **(A**, **B**, **D)**, or DIC are shown. Time-lapse observation was performed for 5 min in **(E)**. Scale bar = 10 µm.

Next, we focused on the morphology of leucoplasts in trichome cells of *parc6* mutants. In WT Arabidopsis plants, leaf trichome leucoplasts are smaller than pavement cell chloroplasts and assume an elongated or irregular shape (Barton et al., 2018). Leaf trichome leucoplasts differentiate from chlorophyll-bearing chloroplasts in the epidermal layer of expanding leaves. As well as at the initiation of trichomes (Barton et al., 2018), rounded chloroplasts were detected during cell growth until the primary branching stage (**Figures 3A**, **B**). Leucoplasts in Arabidopsis trichomes were also examined in the *atminE1* mutant and *AtMinE1* overexpressor line in our previous study (Fujiwara et al., 2009b), which was the only study that observed trichome leucoplasts in chloroplast division mutants.

Consistent with the earlier observation of Barton et al. (2018), trichome leucoplasts in WT Arabidopsis plants emitted no detectable chlorophyll autofluorescence (**Figure 3C**). A single giant mature trichome cell in the upper petiole (and at the junction between petiole and lamina) possibly contained over 100 leucoplasts, although accurate counting was not possible. This number is much larger than the number of leucoplasts in an early developing trichome cell (~30 in **Figure 3B**). The leucoplasts were distributed over the entire trichome cell, including both stalk and branch regions (Folkers et al., 1997). A subset of trichome leucoplasts showed a single or multiple constriction(s) (**Figure 3C**). Some of these plastid constrictions seemed stable and were unaffected by the shape change of the whole organelle (data not shown). Trichome leucoplasts were often found to produce longer stromules than pavement cell chloroplasts. Sometimes trichome leucoplasts surrounded the nucleus (**Figure 3D**), a phenomenon known for chloroplasts in normal pavement cells. Although we were able to determine the location of the nucleus using bright field illumination, fluorescence visualization of the nucleus (CFP) and leucoplasts (YFP) in the FL6-4 transgenic line facilitated more reliable identification of both organelles (**Figure 3D**). Another notable feature of trichome leucoplasts was the amoeba-like deformation. **Figure 3E** shows the process of shape change that occurred within 5 min. Consistent with this observation, we found various shapes of leucoplasts in trichome cells, such as spherical, ovoid, filamentous, amoeboid, dumbbell-shaped, and multiple-arrayed forms (**Figures 3C**–**F**).

Next, we examined the morphology of trichome leucoplasts in *parc6* mutants (**Figures 3G**–**J**, **S2B–D**). The following three alterations in plastid morphology were common to the trichomes of *parc6-1*, *parc6-3*, *parc6-4*, and *parc6-5* mutants. First, grape-like clusters of plastids were detected in *parc6* trichomes, most of which were more highly developed than those in *parc6* pavement cells. We frequently observed the grape-like clusters juxtaposed against the cell nucleus (**Figure 3J**), implying a possible link between the behavior of the nucleus (Mathur et al., 1999) and formation and/or location of the plastid clusters. Nevertheless, this was negated by the presence of clusters in the branch and stalk regions of trichomes (**Figures 3H, I**) and at a distant location from the nucleus (**Figure S2C**). Generally, within a grape-like cluster, plastids showed three-dimensional aggregation (**Supplementary Movies 1**, **2**). Although plastids within a cluster showed a wide variability in size, their shape was relatively uniform and spherical, as revealed by the image from a slightly squashed trichome cell (**Figure S2B**) and two-dimensional projection image of a plastid aggregate (**Figure S2D**). Second, leucoplasts in *parc6* trichomes exhibited a striking formation of stromules. Longer exposure time than that required to obtain images of grape-like clusters revealed extended stromules (**Figures 3G**, **S2C**). Third, the maximum diameter of the main body of a leucoplast in *parc6* trichomes was approximately 5 µm (e.g., 5.1 µm in **Figure 3H** and 4.8 µm in **Figure S2C**). Previously, we defined giant plastids as plastids with a diameter exceeding 6 µm (Fujiwara et al., 2018). In *parc6* trichomes, we did not find spherical leucoplasts that met the criterion for giant plastids. However, *parc6* trichome cells contained filamentous leucoplasts over 6 µm in length, as if their entire bodies were stromules themselves.

Next, we took a closer look at the occurrence of grape-like plastid clusters in *parc6* trichomes. We counted the number of trichomes with and without the clusters in leaf petiole-blade regions. Among the 60 trichomes examined in *parc6-1*, 48 trichomes contained the grape-like plastid clusters. In the other *parc6* mutants, the frequency of clusters in trichomes was even higher: 100% (50/50) in *parc6-3* and *parc6-5* mutants and 98% (49/50) in *parc6-4*. In WT trichomes, the frequency of clusters was 8% (4/50). Because plastids constituting a “cluster” in WT were observed around the nucleus and showed less dense aggregation than those in *parc6* mutants (data not shown), the “cluster” in WT trichomes probably originated from the preferential localization of plastids at the periphery of the nucleus (e.g., Köhler and Hanson, 2000; Kwok and Hanson, 2004; Delfosse et al., 2016) and hence was considered to be a qualitatively different entity than the true cluster in *parc6*. Therefore, the formation of the grape-like plastid clusters in trichomes was recognized as a major cytological characteristic of *parc6* mutants. This was in contrast to the *parc6* pavement cells, where the occurrence of clusters was more sporadic. The number of vesicular plastids per cluster in *parc6* trichomes widely varied from 12 (**Figure 3I**) to approximately 200 (**Figure S2B**). In contrast to those plastid phenotypes, mitochondria in *parc6* trichomes appeared similar to those in WT trichomes (**Figures S2A**, **D**).

### Plastids in Guard Cells of *parc6* Mutants

Next, we examined plastid morphology in the guard cells of *parc6* mutants. In WT Arabidopsis leaves, chloroplasts in guard cells are smaller and less developed than those in pavement cells (Pyke and Leech, 1994; Barton et al., 2016), and there are little differences in chloroplast morphology, number, and pigmentation among guard cells. In some mesophyll chloroplast division mutants such as *arc3*, *arc5*, *arc6*, and *atminE1*, the morphology of plastids in guard cells was clearly distinct from that of plastids in mesophyll cells and pavement cells of the same plants. Moreover, guard cell plastids in these mutants were also unique with respect to the phenotypic differences among individual cells; while guard cell plastids showed diverse morphology among cells within a single tissue sample, mesophyll chloroplasts were relatively uniform. For instance, in the Arabidopsis mutant of *ARC6*, which encodes a key regulator protein of chloroplast division in mesophyll cells, occasional lack of chloroplasts (Robertson et al., 1995) and occurrence of non-photosynthetic plastids (Chen et al., 2009) were observed in guard cells. We previously showed that these non-photosynthetic plastids in *arc6* guard cells proliferate and elongate vigorously (Fujiwara et al., 2018).

**Figure 4:**
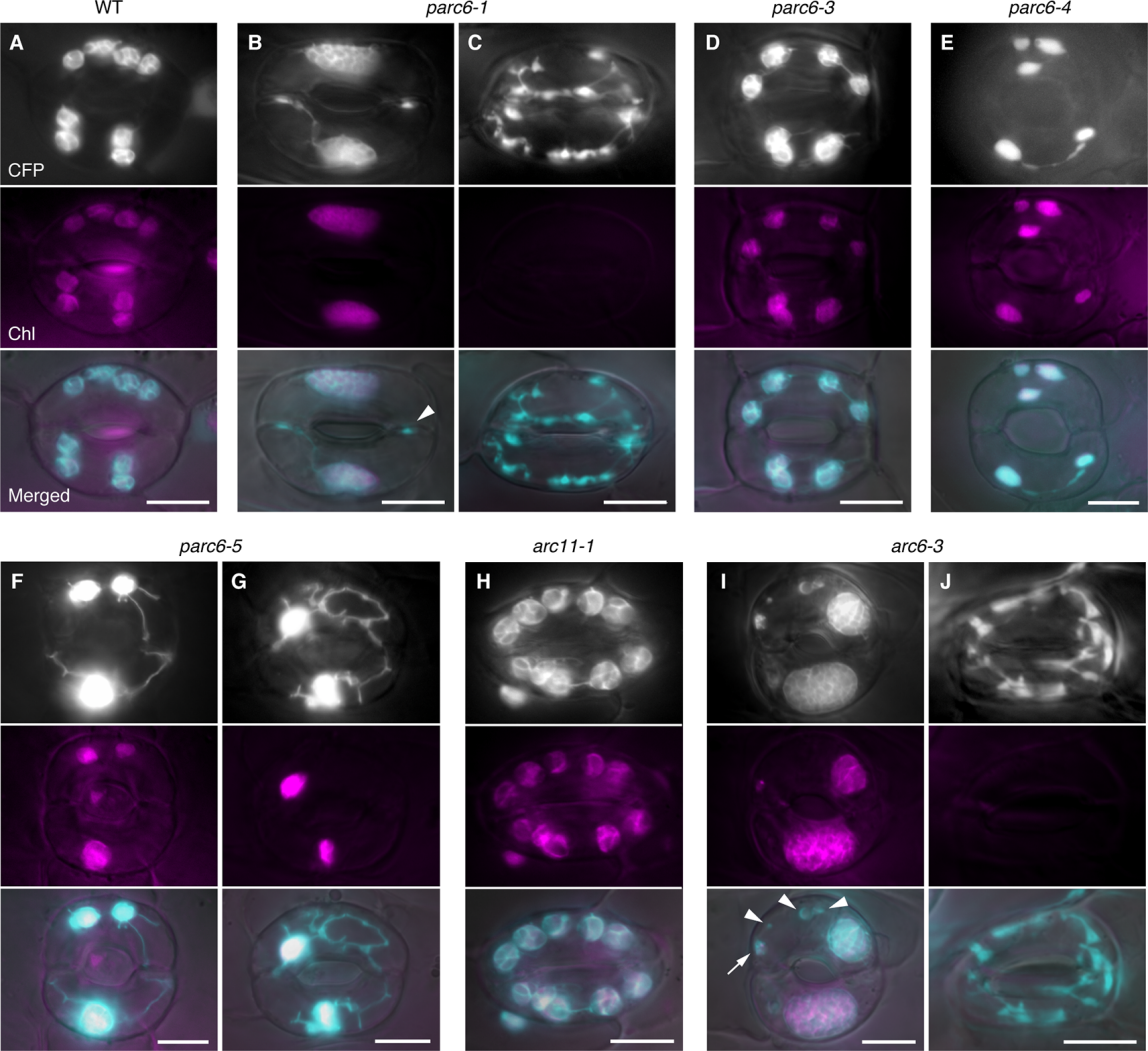
Morphology of plastids in leaf stomatal guard cells of *parc6* and other plastid division mutants. **(A**–**J)** Images of guard cells in the 3^rd^ and 4^th^ leaf petioles of 4-week-old WT **(A)**, *parc6-1* **(B**, **C)**, *parc6-3* **(D)**, *parc6-4* **(E)**, *parc6-5* **(F**, **G)**, *arc11-1* **(H)**, and *arc6-3* **(I**, **J)** seedlings. Fluorescence images of stroma-targeted CFP (black-and-white or cyan-colored in ‘merged’ panels), chlorophyll (magenta), and merged images of CFP/YFP, chlorophyll and DIC are shown. Arrow and arrowheads indicate plastids with and without chlorophyll, respectively. Scale bar = 10 µm.

In accordance with our earlier work, guard cell chloroplasts in the WT were relatively uniform in size and shape and often produced stromules (**Figure 4A**). On the contrary, guard cell plastids in *parc6-5* and other *parc6* mutants displayed variable phenotypes (**Figures 4B**–**G**). The first phenotype was the decrease in number and a complementary increase in size of chloroplasts (**Figures 4B**, **F**, and **G**), a characteristic of mesophyll cell chloroplasts in *parc6*. The increase in chloroplast size in *parc6* guard cells was generally more modest than that in *parc6* pavement cells (**Figures S3A**, **2B**–**G**). The second phenotype was the existence of normally sized and shaped chloroplasts in *parc6* guard cells (**Figures 4D**–**F**). The third phenotype was the hyperproduction of stromules (**Figures 4B**, **F**, **G**), which is typically seen in pavement cells of mutants in which chloroplast division in mesophyll cells is severely inhibited. The extent of stromule development in guard cells was more limited than that in pavement cells as a whole (**Figures 4B–G**, **S3A**, and **B** vs. **Figures 3B–G**). The fourth phenotype was the formation of poorly developed plastids, devoid of chlorophyll autofluorescence (arrowheads in **Figures 4B**, **I**). The fifth and final phenotype was the disappearance of chloroplasts from guard cells (**Figure 4C**), which is typically seen in guard cells of mutants in which chloroplast division in mesophyll cells is severely inhibited. The above five features were commonly observed among all *parc6* mutants used in this study. The type(s) of feature observed in a given guard cell seemed to be independent of the location of the guard cell in the entire leaf and rather appeared to be selected randomly (**Figure S3A**). Additionally, the normally sized and shaped chloroplasts observed in *parc6* guard cells (second phenotype) sometimes displayed more prominent formation of stromules than those in WT guard cells. We also noticed that *parc6* guard cells without chloroplasts (fifth phenotype) were identical to class III guard cells, defined previously as guard cells containing “populations of numerous minute plastids, which were colorless (chlorophyll-less)” (Fujiwara et al., 2018), observed in guard cells of *arc5*, *arc6*, and *atminE1* mutants. In some cases, such colorless class III guard cells in *parc6* mutants outnumbered chloroplasts in WT guard cells on a per cell basis, although accurate counting of plastids in *parc6* guard cells was not feasible owing to their intricate morphology (**Figure 4C**). In *parc6*, we did not find class IV guard cells, defined as guard cells possessing “web-like structures consisting of chlorophyll-less plastids” (Fujiwara et al., 2018). No differences were detected in the morphology of mitochondria between WT and *parc6* guard cells, based on the fluorescence signal of mitochondrion-targeted YFP (**Figure S3A**), similar to the mitochondria in pavement cells (Itoh et al., 2018).

**Figure 5:**
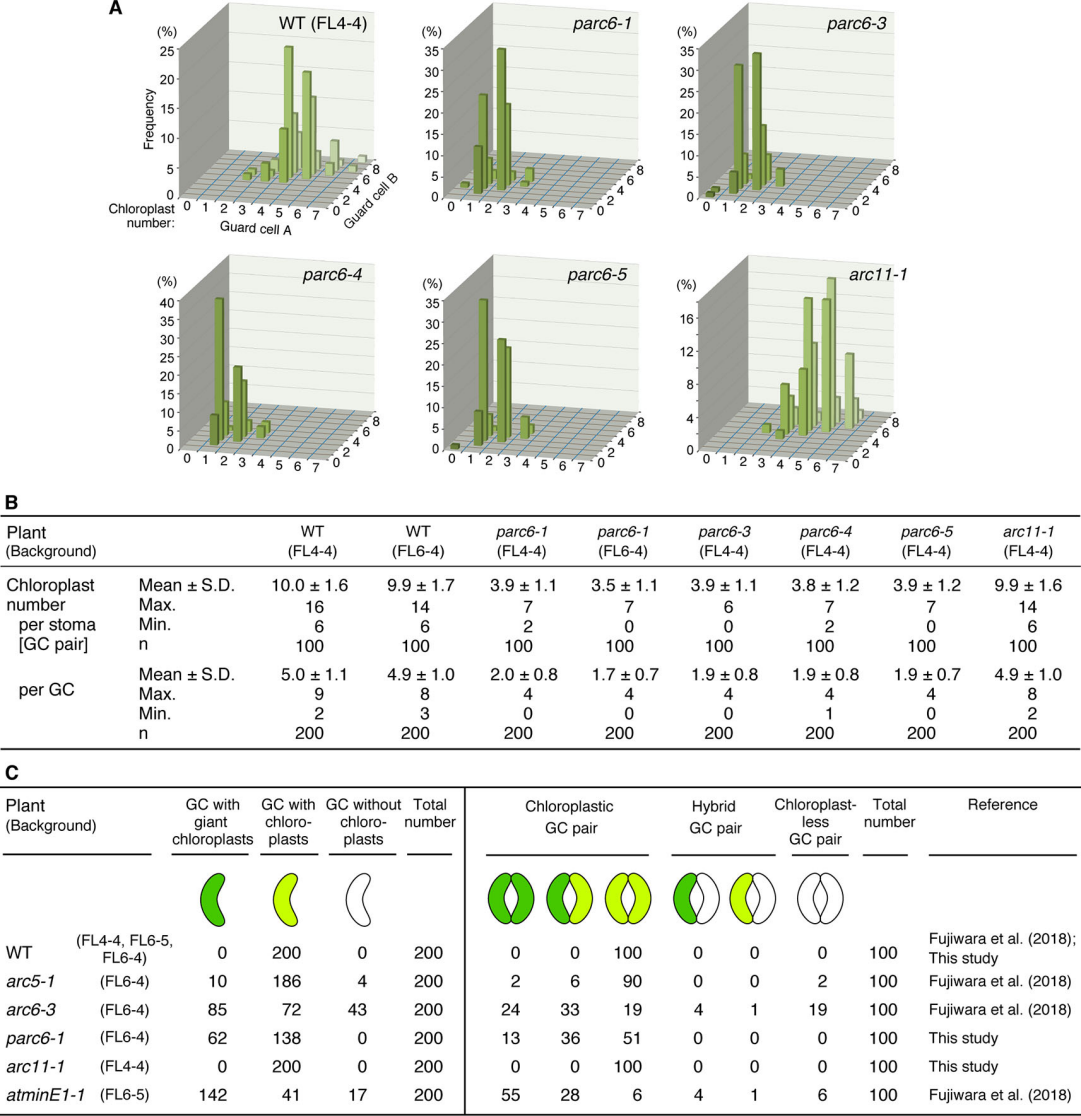
Distribution of chloroplasts in leaf stomatal guard cells of Arabidopsis *parc6* and other plastid division mutants. **(A**, **B)** Measurements of chloroplast number in arbitrarily selected 100 guard cell (described as “GC” in this figure) pairs (“cell A” and “cell B”) of WT, *parc6-1*, *parc6-3*, *parc6-4*, *parc6-5* and *arc11-1* seedlings in graph **(A)** and table **(B)** form. All plants were derived from the FL4-4 line, and the 3^rd^ and 4^th^ leaf petioles of 4-week-old seedlings were examined. **(C)** Frequency of guard cells with giant, normal-sized, or no chloroplasts in *parc6-1*, *arc11-1*, and other mutant plants, based on results in the present study and previous study (Fujiwara et al., 2018). The fluorescence assays were representative of several measurements. Three to five independent leaves were used for each measurement.

Next, we attempted to quantify and compare the phenotypes of guard cell plastids among different *parc6* mutants. We counted the number of chloroplasts in guard cells, both on a per stoma basis (i.e., pair of guard cells) and per guard cell basis. Among the four different *parc6* mutants examined, the number of chloroplasts (both per stoma and per guard cell) was similar (**Figures 5A**, **B**). Plastid partitioning between paired guard cells of each stoma was also similar among the *parc6* mutants (**Figures 5A**, **B**). The mean chloroplast number per guard cell was 5.0 in the WT (FL4-4 line) and ranged from 1.7 to 2.0 in *parc6* mutants. Furthermore, the number of chloroplasts per guard cell ranged from 0–4 among all *parc6* mutants but from 2–9 in the WT. Guard cells devoid of chloroplasts in *parc6* mutants occurred at a frequency of <2%; in a few measurements of *parc6-1* and *parc6-4* mutants, no chloroplast-lacking guard cells were detected (**Figures 5A**, **B**). Additionally, we generated two independent lines using the T-DNA insertion (knockout) mutant *parc6-1*; in one of these lines (*parc6-1* × FL4-4), plastid stroma was labeled with CFP, whereas in the other line (*parc6-1* × FL6-4), plastid stroma was labeled with YFP. Both of these lines showed similar chloroplast number per stoma and per guard cell (**Figure 5B**, **Table 1**). This further supports that the defective allele of *PARC6*, and not the other effects brought on by crossing, is responsible for the decrease in chloroplast number per guard cell and occasional occurrence of chloroplast-devoid guard cells. The results of counting (**Figures 5A**, **B**) also verified that the plastid phenotype observed in *parc6-5*/*suba2* leaf epidermis (Itoh et al., 2018) is quantitatively equivalent to that observed in the other known *parc6* mutants.

Since the guard cell plastid phenotypes were similar among *parc6* mutants and not affected by the fluorescently labeled transgenic line used in crossing, we chose the progeny of *parc6-1* × FL6-4 as a representative *parc6* line in the following experiment. Indeed, we confirmed that the morphology of YFP-labeled guard cell plastids in *parc6-1* × FL6-4 progeny was equivalent to that of CFP-labeled guard cell plastids in *parc6-1* × FL4-4 progeny (**Figure S3B**). We classified guard cells based on the length of guard cell chloroplasts (**Table 2**, **Figure 5C**). Taking into account our previous data on *arc5*, *arc6*, and *atminE1* mutants (Fujiwara et al., 2018), we conclude the following: 1) with regard to the frequency of guard cells containing giant chloroplasts, *parc6* was relatively similar to *arc6* and, to a lesser extent, to *atminE1* (**Figure 5C**); 2) with regard to the frequency of guard cells without chloroplasts, *parc6* was relatively similar to *arc5* (**Figure 5C**); and 3) with regard to the length variation of guard cell chloroplasts, *parc6* showed an intermediate phenotype between *arc5* (shorter guard cell chloroplasts) and *arc6*/*atminE1* (longer guard cell chloroplasts) (**Table 2**).

**Table 2:** Measurement of chloroplast length in leaf stomatal guard cells of WT and *parc6-1* seedlings^1^.

Chloroplast characteristics	WT (FL6-4)	*parc6-1* (FL6-4)
Frequency of chloroplasts		
Giant chloroplast	0 (0.0%)	30 (30.0%)
Normal-sized chloroplast	100 (100.0%)	69 (69.0%)
Mini-chloroplast	0 (0.0%)	1 (1.0%)
Chloroplast length (µm)		
Mean ± standard deviation	4.3 ± 0.7	5.4 ± 1.8
Maximum	5.6	10.9
Minimum	2.6	1.9
Total number of chloroplasts examined	100	100

^1^For details of the experiment, see Materials and Methods.

### Plastids in Trichome and Guard Cells of *arc11* Mutants

Arabidopsis *arc11* is a loss-of-function mutant of *AtMinD1*; in *arc11*, the level of AtMinD1 protein is greatly reduced, and the mutant AtMinD1 protein (A296G) does not localize at the division site or at punctate structures in the chloroplasts, unlike the WT MinD1 protein, as shown by immunofluorescence microscopy (Nakanishi et al., 2009). The mesophyll cells of *arc11* and *parc6* mutants display an abnormal spatial control of stromal FtsZ ring formation, resulting in variable sized chloroplasts within a single cell (Marrison et al., 1999; Fujiwara et al., 2008; Glynn et al., 2009; see **Figure S4**). Previously, we reported the plastid phenotype in pavement cells of *arc11* (Fujiwara et al., 2017; Itoh et al., 2018). To gain further insight into the functional difference between the mesophyll chloroplast FtsZ ring positioning factors PARC6 and MinD1 and its tissue-dependency, we examined the phenotype of plastids in trichome and guard cells of *arc11* using the *arc11-1* × FL4-4 line, whose plastids could be visualized with fluorescence from stroma-targeted CFP. The trichomes of *arc11-1* contained leucoplasts, which were almost indistinguishable from the leucoplasts in trichomes of the WT, in terms of the size, shape, subcellular distribution, and chlorophyll autofluorescence signal (**Figure 3**; data not shown). Similarly, chloroplasts in guard cells of *arc11-1* were almost indistinguishable from those in guard cells of the WT (**Figure 4H**). To verify this observation quantitatively, we measured the morphological traits of guard cell plastids in *arc11* using the same method as that used to measure the guard cells of *parc6* mutants (**Figure 5**). The chloroplast number per stoma and per guard cell and guard cell classification, based on the presence or absence of plastids (**Figure 5**), reinforce our view that *arc11-1* is almost indistinguishable from the WT, at least with respect to the guard cell plastid phenotype.

### Ultrastructural Analysis of Guard Cell Plastids in *parc6*

To reveal more detailed structural features of plastids in the leaf epidermis of *parc6*, we performed TEM analysis. Previously, we reported the TEM images of chloroplasts in mesophyll and pavement cells of *parc6* mutants (*parc6-3* and *parc6-5*) (Itoh et al., 2018). In the present study, we focused on the ultrastructure of plastids in *parc6* guard cells. Generally, guard cells are smaller than mesophyll and pavement cells; therefore, the chloroplast density is relatively higher in guard cells than in mesophyll and pavement cells. We expected that this feature of guard cells would be more advantageous for TEM analysis of the whole structure of the variable and complexly shaped *parc6* plastids (**Figure 4**), which had been a difficult task for mesophyll and pavement cells (Itoh et al., 2018). We also compared the plastid ultrastructures of different plant lines (WT and *parc6* ^−/−^ backgrounds) with and without the transgene expressing the stroma-targeted fluorescent protein. To the best of our knowledge, no studies have reported a secondary effect of stroma-localized fluorescent protein on plastid ultrastructures in any plastid division mutants. In this experiment, we used *parc6-1* (Col background) and *parc6-5* [a mutant obtained from an EMS-mutagenized population of FL4-4 (Col background), in which plastids and mitochondria are labeled with CFP and YFP, respectively].

**Figure 6:**
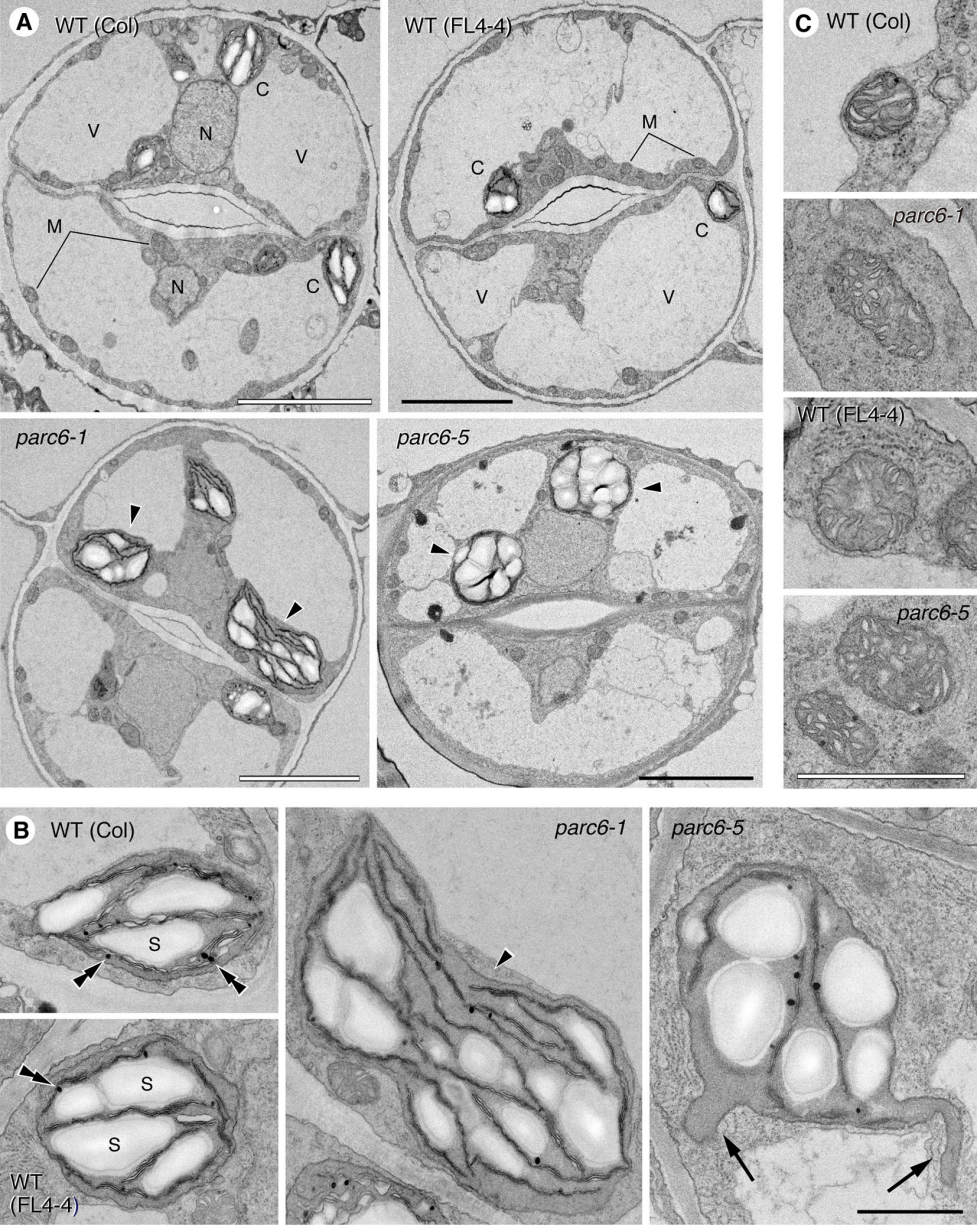
Ultrastructure of plastids and mitochondria in leaf stomatal guard cells of *parc6* mutants. **(A)** Guard cells. **(B)** Plastids. **(C)** Mitochondria. Adaxial epidermis of the first leaf of 10-day-old WT (Col), WT (FL4-4 line), *parc6-1* (Col-derived), and *parc6-5* (FL4-4-derived) seedlings. Arrowheads, arrows, and double arrowheads indicate enlarged chloroplasts, stromules, and plastoglobules, respectively. Scale bars: 5 µm **(A)**; 1 µm **(B**, **C)**.

We examined guard cells in the adaxial surface of petioles of the first and second leaves of 10-day-old seedlings of *parc6-1* and *parc6-5* mutants and their respective parental strains, WT (Col) and WT (FL4-4). All four lines showed the previously described characteristics of cellular and subcellular structures of guard cells (Zhao and Sack, 1999), except for plastids in *parc6* mutants (**Figure 6A**). The two symmetrical guard cells showed a large central vacuole(s) occupying most of the cell volume, cell nucleus located near the stomatal aperture, and mitochondria distributed throughout the cytoplasm in all lines. Additionally, chloroplasts in all lines showed well-developed starch grains in the stroma, less organized thylakoid membranes with few grana stacks, and plastoglobules (**Figure 6B**). Comparison between WT (Col) and WT (FL4-4) lines showed no major difference in the size, shape, and internal structure of chloroplasts. Similarly, both *parc6-1* and *parc6-5* mutant lines contained variably sized chloroplasts (normal to giant), within a similar size range. Another characteristic of the *parc6* guard cell plastids was the frequent formation of stromules, surrounded by two envelope membranes (**Figure 6B**). The size and internal structure of mitochondria also showed no major differences among the four lines examined (**Figure 6C**), which was consistent with the fluorescence microscopy images (**Figure S3A**).

### Localization of PARC6-GFP in Petiole Cells

Lastly, we investigated the subcellular localization of PARC6 using GFP as a reporter. Glynn et al. (2009) demonstrated the complementation of the Arabidopsis *parc6-1* mutant with the WT *PARC6* transgene fused to *GFP* gene under the control of the constitutive CaMV35S promoter and intraplastidic localization of PARC6-GFP in young leaf epidermal cells in this complementation line. Nonetheless, it remains elusive how PARC6 behaves during the entire process of chloroplast division. In the present study, we constructed a chimeric construct consisting of the upstream region of the *PARC6* gene, full-length *PARC6* cDNA, and *GFP* and introduced this construct into Arabidopsis *parc6-1* and *parc6-4* mutants. This construct was able to fully rescue the division defect in both *parc6* mutants (data not shown). Using these complementation lines, we observed cortical and epidermal cells in leaf petioles, which correspond to mesophyll and pavement cells in leaf blades, respectively.

**Figure 7:**
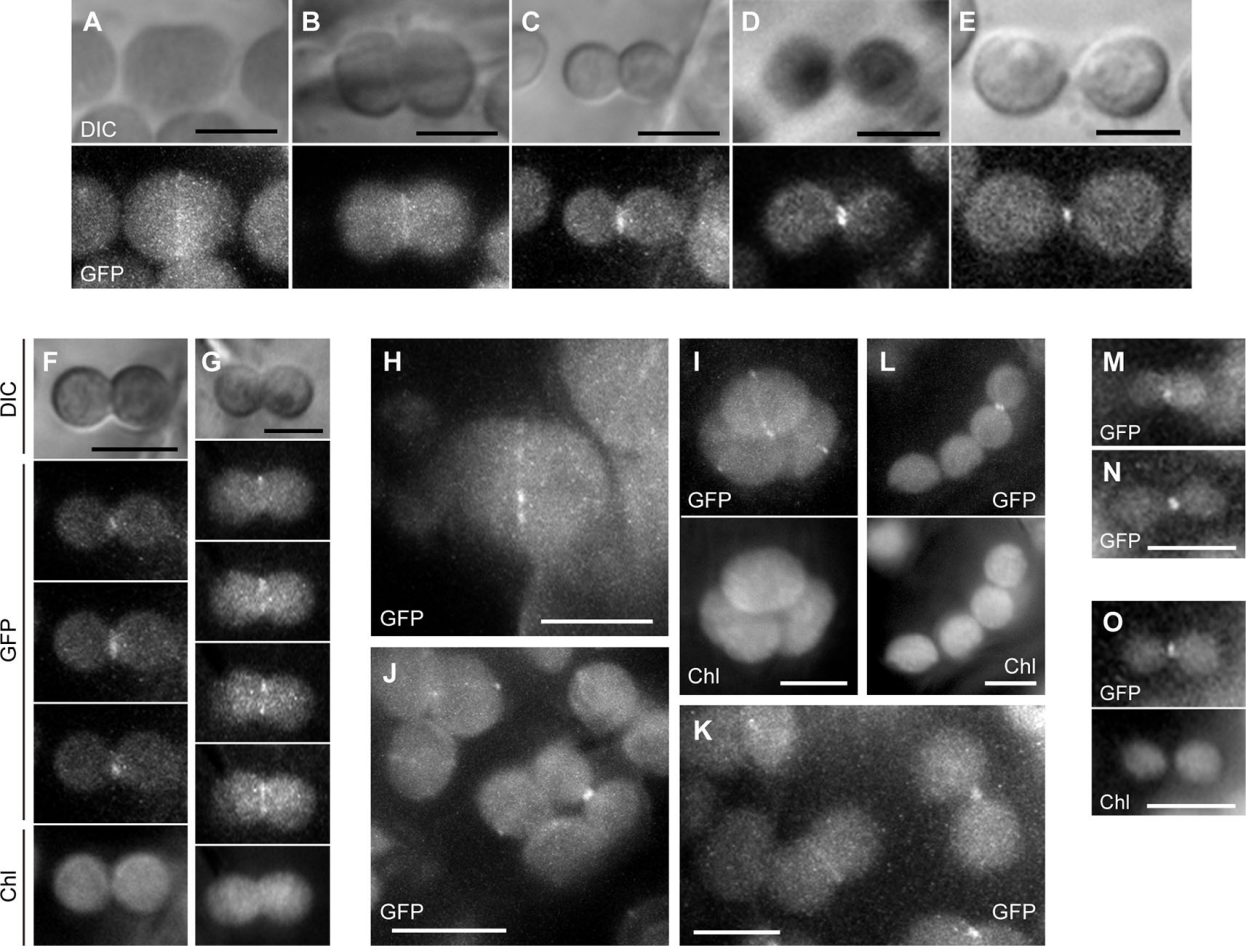
Analysis of PARC6-GFP localization in leaf cortex and pavement cells. **(A**–**O)** Images of chloroplasts in leaf petioles of 1–3-week-old seedlings of *parc6-1* complementation lines. Images of full-length PARC6-GFP, chlorophyll, or DIC in cortex **(A**–**K)** and pavement cells **(M**–**O)** are shown. Scale bars: 10 µm **(J)**; 5 µm (others).

In the *parc6-1* complementation line, PARC6-GFP was localized at the constricting neck of dividing chloroplasts as a filamentous or punctate pattern in cortex cells starting from the initial to the final stages of chloroplast division (**Figures 7A**–**E**). When we shifted the focal plane of the microscope from the top to the bottom of dumbbell-shaped chloroplasts at the middle stage of division, the GFP signal appeared as a filament over the constricting neck in the top and bottom focal planes but as two dots at opposite sides of the neck in the intermediate plane (**Figures 7F**, **G**). At the final stage of chloroplast division, the GFP signal was detected as a single focus (**Figure 7E**). These data suggest that PARC6 forms a ring surrounding the constricting neck of dividing chloroplasts. Moreover, at the early stage of chloroplast division, the GFP signal appeared as short filaments aligned discontinuously, like a dashed line, along the equatorial division plane of chloroplasts (**Figures 7H**, **I**). On the contrary, at later stages when chloroplasts were more deeply constricted, the GFP signal was detected as a continuous filament at the same position (**Figures 7B**–**D**). These observations imply that during the early to middle stages of constriction formation in dividing chloroplasts, PARC6 ring changes its configuration from a discontinuous array of short fragments to a continuous ring, possibly by gradual polymerization of PARC6 initiated at multiple sites along the circumference of the constriction. We further compared the signal intensities of the ring-like structures of PARC6 at different stages of chloroplast division under the same conditions of excitation and image acquisition (**Figures 7J**, **K**). The GFP signal was faint at the early stage, modest at the intermediate stage, and relatively strong at the late and final stages. This suggests that as chloroplast division proceeds, the PARC6 ring becomes denser by maintaining the number of PARC6 proteins within it, despite the progressive decrease in its diameter. Even after the complete separation of chlorophyll autofluorescence derived from the thylakoids of daughter chloroplasts, the PARC6 ring persisted at the original neck region between them (**Figure 7L**). The PARC6 rings were often detected between two attached but apparently separate chloroplasts, based on the bright field images. **Figures 7M–O** show the localization of PARC6-GFP in chloroplasts of the epidermal cells. Because the chloroplasts in the epidermis were immature, detailed tracing of the behavior of PARC6 during chloroplast division was difficult. Nevertheless, the results (**Figures 7M–O**) were consistent with the abovementioned results of the cortex cells and thus support the notion that the localization pattern and behavior of PARC6 are largely conserved between cortex and epidermal cells. Additionally, the present results of epidermal cells were also consistent with the earlier report (Glynn et al., 2009). We also examined the *parc6-4* complementation line in the same manner as the *parc6-1* complementation line and obtained similar results (**Figure S5**; only a subset of data is shown). Taken together, these data indicate that the ring-like structure of PARC6 changes its configuration with the progression of chloroplast division.

## Discussion

### Probable Causal Mutation in *parc6-5*/*suba2*

In this study, we further characterized the *parc6-5*/*suba2* mutant (Itoh et al., 2018) and other *parc6* alleles of Arabidopsis, while focusing on the role of *PARC6* in morphology maintenance of non-mesophyll plastids in the leaf epidermis. First, the effect of G62R, one of two mutations in *parc6-5*, on the plastid protein import efficiency was evaluated (**Figure 1**). In model plant species such as Arabidopsis, transient expression of a reporter gene, such as *GUS* and *GFP*, can be visually detected usually within 2 to 4 days after plasmid delivery using biolistics (Ueki et al., 2009). In our experiments, no difference was detected in the spatial distribution of fluorescence signals in cells (particularly in terms of the accumulation in plastids and staying in the cytoplasm) between TP-GFP and TP_G62R_-GFP, irrespective of the types of bombarded cells, including tobacco pavement and guard cells containing chloroplasts and onion bulb epidermal cells containing leucoplasts, at the relatively early time points (6–24 h post-bombardment) (**Figures 1B**, **C**, **E**, **F**). It is possible that at later time points, saturated GFP signal from plastids would mask the putative difference in the protein import rate. Our results at 6–24 h indicate therefore that TP_G62R_ functions as efficiently as the WT TP. Thus, by a process of elimination, it is likely that the W700stop nonsense mutation is responsible for the phenotype of *suba2*/*parc6-5*. Accordingly, it was suggested that the C-terminal 120-amino acid region (amino acids 700–819) of PARC6 is critical for its function. This conclusion is in agreement with an earlier report that showed a chloroplast division defect in another Arabidopsis *parc6* mutant, *arc6h* (Ottesen et al., 2010), harboring a frameshift mutation, causing V697stop mutation and eventually a C-terminal truncation of PARC6, quite similar to that in *parc6-5*. The C-terminal 223-amino acid region (amino acids 597–819) of PARC6 is exposed in the intermembrane space, as shown by biochemical analysis, which facilitates the interaction of PARC6 with PDV1, as shown by the yeast two-hybrid assay (Zhang et al., 2016). PDV1 and its paralog PDV2 are outer envelope membrane proteins of plastids, which recruit a dynamin-related protein DRP5B (ARC5) from the cytosol to the plastid surface (Miyagishima et al., 2006). Our results suggest that C-terminal 120-amino acid region of PARC6 exposed within the intermembrane space is indispensable for its function, probably because of its association with PDV1 and the resultant recruitment of DRP5B, although precise functions of PDV1 and DRP5B and their physical and functional association with PARC6 are currently unknown in non-mesophyll plastids.

### Significance of *PARC6* in Plastid Replication and Morphology Maintenance in the Leaf Epidermis

Building on our previous results (Itoh et al., 2018), but by examining younger leaves of *parc6* mutants, we obtained additional insights into the involvement of *PARC6* in chloroplast morphology maintenance in pavement cells. The most remarkable discovery was the occurrence of the grape-like plastid clusters in pavement cells (**Figures 2D**–**G**, **S1C**). Similarly, in trichome cells, the occurrence of the grape-like clusters was the most striking cytological feature of *parc6* mutants (**Figures 3G**–**J**, **S2B**–**D**). This is the first report of the three-dimensional structure of plastid clusters (**Supplementary Movies 1**, **2**). Furthermore, to the best of our knowledge, this is the first report describing the morphology and distribution pattern of plastids (leucoplasts) in the trichomes of any chloroplast division mutants. Overall, in *parc6* mutants, the phenotype of plastids in trichomes (**Figure 3**) was unique compared with that of plastids in mesophyll cells, pavement cells, and guard cells. We believe that the data reported in this study offer a basis for probing the mechanisms of replication and morphology maintenance (including stromule formation) in non-mesophyll plastids and the change of mechanisms during cell differentiation, which are largely unknown at present (Pyke, 2010; Pyke, 2013; Pyke, 2016). Since the developmental process of trichomes in Arabidopsis is well understood and can be readily traced because of their large size and unicellular nature (Hülskamp, 2019), trichomes might be a potential experimental system for resolving the above issues.

The present study showed a significant decrease in the number of chloroplasts in guard cells of *parc6* mutants (**Figures 5A**, **B**). Previously, we performed similar analyses on other chloroplast division mutants including *arc5*, *arc6*, and *atminE1* (F_2_ siblings derived from a cross between the original mutant and FL6-4 or FL6-5) (Fujiwara et al., 2018) and presented the results of guard cell chloroplast counting as integer values, which makes the direct comparison with the present results of *parc6* mutants difficult. When compared in a unified manner, the chloroplast numbers on a per guard cell basis were as follows: WT, 4.6 ± 1.0, max. 7, min. 3 [5 ± 1 in Fujiwara et al., 2018 (the same hereafter)]; *arc5-1*, 3.9 ± 1.3, max. 9, min. 0 (4 ± 1); *arc6-3*, 2.5 ± 1.6, max. 7, min. 0 (2 ± 2); *atminE1-1*, 2.6 ± 1.3, max. 6, min 0. (3 ± 1); *parc6-1*, 1.7 ± 0.7, max. 4, min. 0. This direct comparison suggests that among the examined genes, *PARC6* exhibits the most effective control of chloroplast number in guard cells. By contrast, phenotypic analysis of these mutants, based on the classification of guard cells by size and presence/absence of chloroplasts in them (**Figure 5C**, **Table 2**), suggests that the importance of *PARC6* is intermediate between that of *ARC6*/*AtMinE1* (higher importance) and *ARC5* (lower importance) in terms of chloroplast morphology and differentiation in guard cells.

During leaf development in WT Arabidopsis plants, chloroplasts in pavement cells undergo symmetric binary fission, as mediated by the FtsZ1 ring formation at the equatorial plane (Fujiwara et al., 2015). By contrast, chloroplasts in *arc11-1* pavement cells exhibited aberrant assembly of the FtsZ1 ring(s) at a non-equatorial site or multiple sites, which is consistent with our previous report (Fujiwara et al., 2017). Nonetheless, chloroplasts that initiated such asymmetric or multiple divisions were likely to complete the division process, eventually assuming a morphology that was largely indistinguishable from WT chloroplasts. Thus, the “terminal” phenotype of pavement cells in mature *arc11* leaves was slight heterogeneity in chloroplast size. Chloroplasts in both trichomes and guard cells of *arc11* appeared similar to those in their WT counterparts (**Figures 3K**, **4H**, and **5**), which further underlines the difference in epidermal plastid phenotype between *arc11* and *parc6*. Although AtMinD1 (ARC11) and PARC6 function in the same process of chloroplast division (namely, division site placement) in mesophyll cells, our previous (Fujiwara et al., 2017; Itoh et al., 2018) and present results suggest that the known role and importance of both proteins are modified and differentiated upon morphogenesis of leaf epidermal plastids. AtMinD1 and PARC6 were previously demonstrated to interact with each other in the yeast two-hybrid system (Itoh et al., 2018). It remains uncertain, however, whether they actually interact with each other *in vivo*, i.e., in chloroplasts and other types of plastids. It is possible that the mode and extent of AtMinD1–PARC6 interaction vary with the type of plastids, thereby giving rise to a variety of mechanisms that control plastid division.

Although *suba2*/*parc6-5* was originally identified as one of the two stromule-overproducing mutants by fluorescence microscopy-based screening of leaf epidermal tissues, we failed to detect stromules in the leaf epidermis at the ultrastructural level in our previous study (Itoh et al., 2018). Here, we presented ultrastructural evidence for the active production of stromules in *parc6* guard cells (**Figure 6B**), substantiating the abovementioned speculation that guard cells are suitable for the ultrastructural analysis of complexly shaped plastids, often accompanied by highly developed stromules, in chloroplast division mutants. At the ultrastructural level, guard cell plastids in *parc6-5* were morphologically equivalent to those in *parc6-1*, further supporting the results of fluorescence microscopy (**Figures 4** and **5A**, **B**). Our TEM investigations of WT and *parc6* plants with and without the expression of stroma-targeted CFP and matrix-targeted YFP (**Figures 6B**, **C**) also address the question whether the accumulation of a fluorescent protein in a particular organelle affects the internal and external structures of that organelle. It is widely believed that GFP and its derivatives are not cytotoxic to plants (Stewart Jr, 2001), although the possible effects of these reporter proteins on organelles remain elusive in plants. Comparison of fluorescent protein-expressing and non-expressing plants in an otherwise identical genetic background (WT or *parc6* ^−/−^ in Col background) unequivocally established that the accumulation of fluorescent protein in the stroma and matrix does not affect the structures of plastids and mitochondria, respectively, at least at a detectable level (**Figures 6B**, **C**).

At present, it remains an open question how a defect in PARC6 leads to a variety of abnormal plastid morphologies in leaf epidermal cells. One possibility is that PARC6 has an epidermis-specific function in plastid morphogenesis, in addition to its established function in the division of mesophyll cell chloroplasts, as discussed in our previous report (Itoh et al., 2018). Another possibility is that an arrest of plastid division due to the lack of functional PARC6 secondarily causes abnormal plastid phenotypes during the differentiation of epidermal cells, but in a different and more complicated manner than in mesophyll cells, which consistently show simple phenotypes (i.e., increase in chloroplast size and decrease in chloroplast number). A prerequisite for the latter possibility is that plastids divide at least once during epidermis development. In flowering plants, cells in the outermost cell layer of the shoot apical meristem, namely, the L1 layer, undergo anticlinal cell division and eventually differentiate into pavement, trichome, and stomatal guard cells constituting the epidermis (Glover et al., 2016). While cells in the shoot apical meristem contain undifferentiated proplastids, pavement, trichome, and guard cells contain poorly developed chloroplasts, leucoplasts converted from chloroplasts, and well-developed chloroplasts, respectively (Pyke, 2009; Barton et al., 2016; Barton et al., 2018). In the pavement cells of Arabidopsis, we have previously shown that peanut-shaped chloroplasts associated with the mid-plastid FtsZ1 accumulation, implying that these chloroplasts are in the process of FtsZ1 ring-mediated binary division (Fujiwara et al., 2015). Leucoplasts in trichomes seemed to increase in number during the development of trichome cells in Arabidopsis, as inferred from a rough comparison between early developing trichomes (containing ~30 leucoplasts; **Figures 3A**, **B**) and mature trichomes (containing >100 leucoplasts; **Figure 3F**), although leucoplasts in trichomes could not be accurately counted in this study because of the difficulty imposed by the extremely large size of trichomes. Supporting this notion, in our preliminary experiments, we observed FtsZ1 ring-associated, constricted leucoplasts in Arabidopsis trichomes (Fujiwara, unpublished data). Similarly, in Arabidopsis guard cells, our data suggest FtsZ1 ring-mediated division of chloroplasts (Fujiwara et al., 2019). We cannot, therefore, exclude the latter possibility mentioned above. To determine how a mutation in *PARC6* leads to abnormal plastid morphology in leaf epidermal cells, further investigation of the *parc6* mutant is needed to understand the processes during which the morphological phenotypes of plastids become apparent along the lineage of each type of epidermal cell and to determine the activity (evaluated by plastid counting) and mode (e.g., symmetric vs. asymmetric, binary vs. multiple) of plastid division in differentiating and differentiated epidermal cells.

### Dynamics of PARC6 at the Plastid Division Site

Previously, a functional PARC6-GFP fusion protein was shown to localize to mid-plastid puncta in ovoid and partially constricted chloroplasts, a mid-plastid spot in deeply constricted chloroplasts, and a polar spot at the surface of round, probably post-dividing chloroplasts, which appeared to be the remnant of mid-plastid spots, in the epidermis of young expanding leaves of 14-day-old transgenic Arabidopsis seedlings (Glynn et al., 2009). In the current study, we employed similar methodology as that used by Glynn et al. (2009) but used both cortical and epidermal tissues in the leaf petioles to provide more detailed information on the subplastidic dynamics of PARC6. The PARC6-GFP proteins localized to the middle of pre-dividing and dividing chloroplasts as well as to a single polar spot in post-dividing chloroplasts (**Figure 7**), as described above. However, we noted two aspects of PARC6 localization: 1) formation of a PARC6 ring surrounding the constricting neck of dividing chloroplasts (**Figures 7A**–**G**), and 2) discontinuous nature of the PARC6 ring at the early stage of chloroplast division (**Figures 7H**, **I**). Although the formation of a PARC6 ring around the dividing chloroplasts was formerly hinted at by Glynn et al. (2009), direct evidence for this has been lacking to date. More importantly, the discontinuous nature of the PARC6 ring early during chloroplast division observed in this study is in contrast to the progressive concentration of PARC6 in a single spot at the isthmus of highly constricted chloroplasts at the final stage of division [Glynn et al., 2009; this study (**Figure 7E**)]. To the best of our knowledge, PARC6 is the first protein reported to form an array of short filaments at the chloroplast division site at the early division stage. Among the known mid-plastid-localizing proteins, DRP5B (ARC5) and PDV1 clearly show a discontinuous, “array-of-dot”-like localization pattern surrounding the division site, as revealed by GFP tagging (for DRP5B and PDV1) and immunofluorescence microcopy (for DRP5B) (Miyagishima et al., 2006; Okazaki et al., 2009; Miyagishima et al., 2011). Components of the chloroplast division site-determining Min system, including AtMinD1, AtMinE1, and MCD1 (Osteryoung and Pyke, 2014), also appear to exhibit a punctate pattern, as revealed by immunofluorescence microcopy (Nakanishi et al., 2009; Fujiwara et al., 2009a; Miyagishima et al., 2011; Chen et al., 2018b). Nevertheless, the observed punctate signals of AtMinD1, AtMinE1, and MCD1 were generally smaller, more varied in size, and more irregularly aligned along the division plane than those of DRP5B and PDV1 (e.g., Figure 4.3 in Miyagishima et al., 2011). The discontinuous localization pattern of PARC6-GFP (**Figures 7H**, **I**) seems to bear a greater resemblance to that of GFP-DRP5B (see Figure 4G in Miyagishima et al., 2006) and GFP-PDV1 (see Figures 4A, C in Miyagishima et al., 2006) in unconstricted chloroplasts rather than to that of AtMinD1, AtMinE1, and MCD1. This might imply partial colocalization of PARC6 with PDV1 and DRP5B in the early stage of chloroplast division. In fact, regions of PARC6 and PDV1 in the intermembrane space were shown to interact with each other in yeast two-hybrid and pull-down assays (Zhang et al., 2016). Additionally, DRP5B and the cytosolic region of PDV1 were also demonstrated to interact with each other by both yeast two-hybrid and bimolecular fluorescence complementation assays (Holtsmark et al., 2013), thereby enabling the recruitment of DRP5B from the cytosol to the outer envelope surface of chloroplasts at the division site (Miyagishima et al., 2006). This suggests that PARC6, PDV1, and DRP5B might act together during a certain period of chloroplast division initiation. However, at a later stage of chloroplast division, when chloroplasts were clearly constricted, GFP-PDV1 and GFP-DRP5B still showed an “array-of-dot”-like localization (see Figures 4D, H in Miyagishima et al., 2006), whereas PARC6-GFP appeared to form a continuous ring in similarly constricted chloroplasts (**Figure 7C**). This highlights the differential dynamics of PARC6 and PDV1/DRP5B upon the constriction of chloroplasts.

In the PARC6 localization experiment, the fluorescence signal of PARC6-GFP in chloroplasts was detected only in young, emerging leaves (shorter in length than several millimeters). Thus, it remains unknown whether the above-described PARC6 dynamics commonly exists in chloroplasts of mesophyll and pavement cells at every developmental stage. Moreover, because the fluorescence signal of PARC6-GFP was quite faint, a long exposure time was needed to capture the GFP signal, making it impossible to obtain GFP images in trichome and guard cells in the current study (not shown). Despite such technical difficulties, change in the expression and possibly localization of PARC6 during leaf development deserves future investigation because GFP-PDV1 (and GFP-PDV2) expressed by their respective promoters were also detected in young, emerging leaves but not in older, expanding leaves (Okazaki et al., 2009), similar to PARC6. Simultaneous labeling and imaging of PARC6 and PDV1 would be particularly important for the elucidation of the assembly process of the plastid division machinery and its possible dependency on the developmental stage and differentiation state of the leaf cell.

## Data Availability Statement

The datasets generated for this study are available on request to the corresponding author.

## Author Contributions

RI designed the study. HI, MY, NK, AS, SM, AN, KM, SS, YK, YH, MF, and RI performed the experiments. HI, MY, NK, MF, and RI interpreted the data. AS, MF, and RI wrote the paper. YK, TA, MF, and RI contributed reagents, materials, and/or analytical tools.

## Funding

This work was supported by the Ministry of Education, Culture, Science and Technology of Japan under KAKENHI (grant nos. 22780087, 25450136, and 19K05831 to MF; 25292009 to YK; and 26440152 and 18K06314 to RI) and by the Uruma Fund for the Promotion of Science (to RI).

## Conflict of Interest

The authors declare that the research was conducted in the absence of any commercial or financial relationships that could be construed as a potential conflict of interest.
