# Supplementary material for: Arabidopsis PARC6 Is Critical for Plastid Morphogenesis in Pavement, Trichome, and Guard Cells in Leaf Epidermis
Source: Front Plant Sci. 2020 Jan 15;10:1665. doi: 10.3389/fpls.2019.01665 (PMC6974557; doi:10.3389/fpls.2019.01665)
Supplement: Supplementary file 1 [file DataSheet_1.pdf]

# *Supplementary Material*

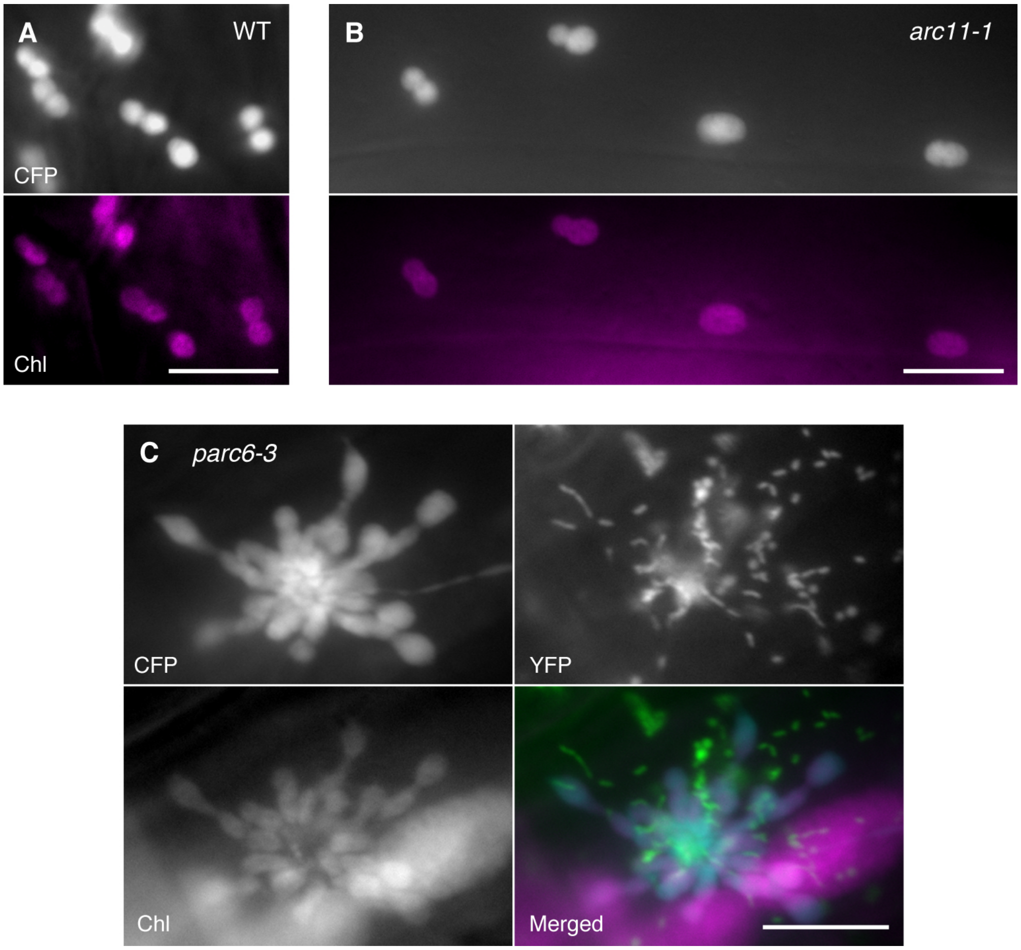


**Supplementary Figure S1.** Morphology of plastids in leaf epidermal pavement cells of *arc11* and *parc6* mutants. **(A–C)** Images of pavement cells in the 3rd and 4th leaf petioles of 2-week-old wild-type (WT) **(A)**, *arc11-1* **(B)** and *parc6-3* **(C)** seedlings. Images of stroma-targeted CFP, matrix-targeted YFP or chlorophyll autofluorescence (chl), and a merged image of CFP (cyan), YFP (green) and chl (magenta) **(C)** are shown. Scale bar = 10 µm.
