# Supplementary material for: Arabidopsis PARC6 Is Critical for Plastid Morphogenesis in Pavement, Trichome, and Guard Cells in Leaf Epidermis
Source: Front Plant Sci. 2020 Jan 15;10:1665. doi: 10.3389/fpls.2019.01665 (PMC6974557; doi:10.3389/fpls.2019.01665)
Supplement: Supplementary file 2 [file DataSheet_2.pdf]

Supplementary Material

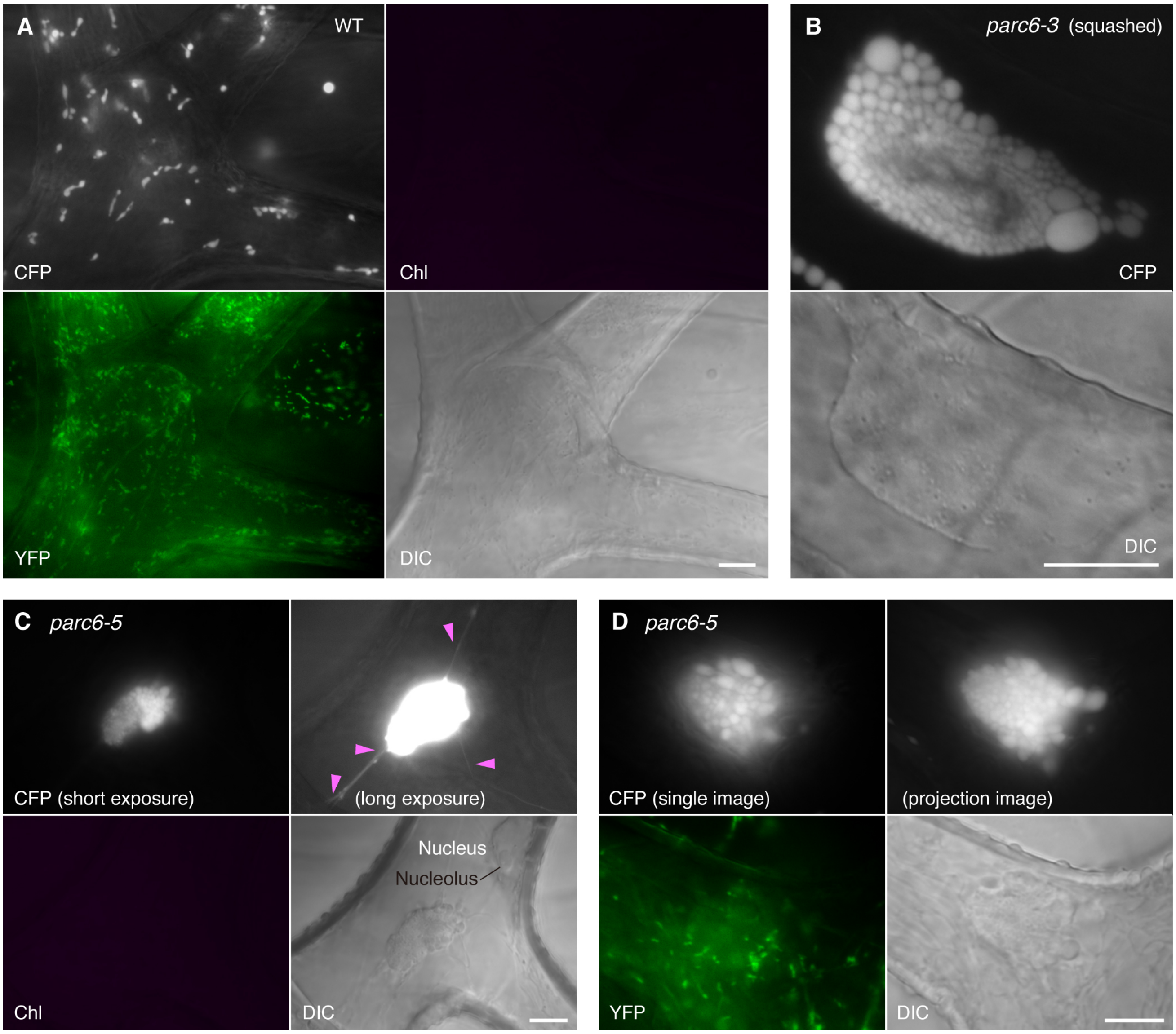

Supplementary Figure S2. Morphology of plastids in leaf trichome cells of *parc6* mutants. **(A–D)** Images of mature trichomes in leaf petioles of WT **(A)**, *parc6-3* **(B)**, and *parc6-5* **(C, D)** seedlings. The 3rd and 4th leaves of 2-week-old seedlings were mainly observed. Images of stroma-targeted CFP, matrix-targeted YFP (green), chlorophyll autofluorescence (magenta) or DIC are shown. Grape-like plastid aggregations were observed in a slightly squashed cell **(B)**, with a different exposure time **(C)**, or at distinct foci **(D)** (a projection image constructed from four images). Arrowheads indicate stromules. Scale bar = 10 µm.
