# Supplementary material for: Arabidopsis PARC6 Is Critical for Plastid Morphogenesis in Pavement, Trichome, and Guard Cells in Leaf Epidermis
Source: Front Plant Sci. 2020 Jan 15;10:1665. doi: 10.3389/fpls.2019.01665 (PMC6974557; doi:10.3389/fpls.2019.01665)
Supplement: Supplementary file 3 [file DataSheet_3.pdf]

Supplementary Material

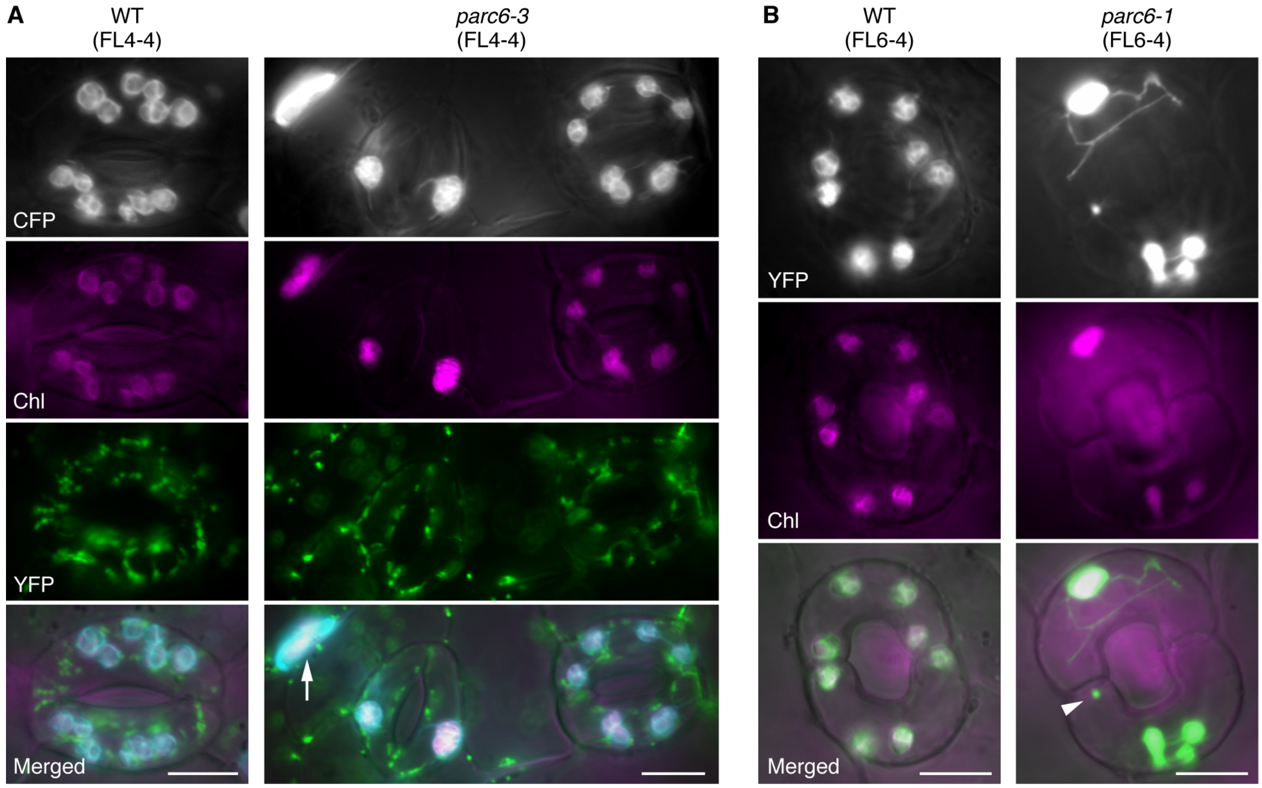

Supplementary Figure S3. Morphology of plastids in leaf stomatal guard cells of *parc6* mutants. **(A, B)** Images of guard cells in 4-week-old WT (FL4-4), *parc6-3* (FL4-4-derived), WT (FL6-4), and *parc6-1* (FL6-4-derived) seedlings. Fluorescence images of stroma-targeted CFP **(A)** or YFP **(B)** (black-and-white in top panels; pseudo-colored in cyan or green in bottom panels, respectively), chlorophyll (pseudo-colored in magenta), and matrix-targeted YFP **(A)** (pseudo-colored in green), and merged images of fluorescence and DIC are presented. Scale bar = 10 µm.
