# Supplementary material for: Arabidopsis PARC6 Is Critical for Plastid Morphogenesis in Pavement, Trichome, and Guard Cells in Leaf Epidermis
Source: Front Plant Sci. 2020 Jan 15;10:1665. doi: 10.3389/fpls.2019.01665 (PMC6974557; doi:10.3389/fpls.2019.01665)
Supplement: Supplementary file 4 [file DataSheet_4.pdf]

Supplementary Material

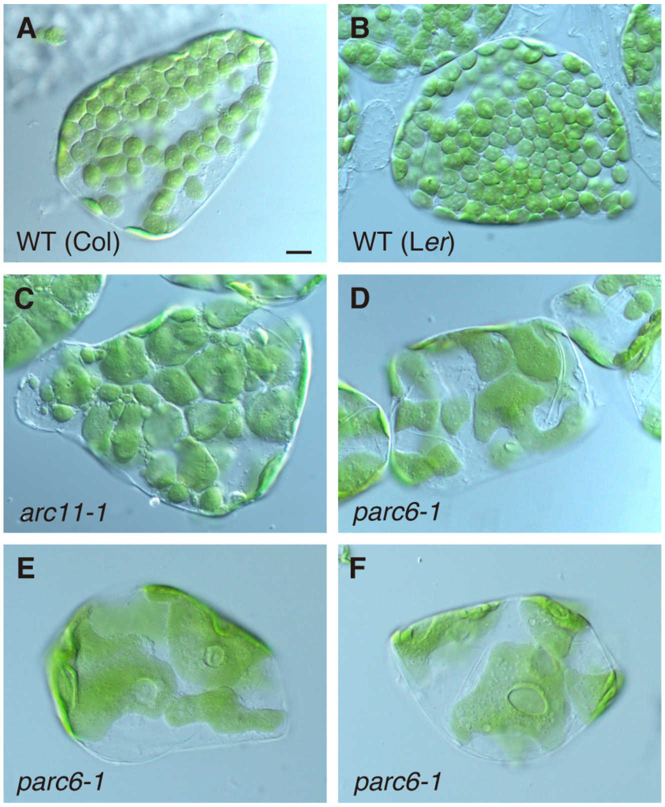

Supplementary Figure S4. Morphology of plastids in leaf mesophyll cells of *arc11* and *parc6* mutants. **(A–F)** Images of mesophyll cells in leaf blades of WT (Col) **(A)**, WT (L*er*) **(B)**, *arc11-1* **(C)**, and *parc6-1* **(D–F)** seedlings. Scale bar = 10 µm.
