# Supplementary material for: Arabidopsis PARC6 Is Critical for Plastid Morphogenesis in Pavement, Trichome, and Guard Cells in Leaf Epidermis
Source: Front Plant Sci. 2020 Jan 15;10:1665. doi: 10.3389/fpls.2019.01665 (PMC6974557; doi:10.3389/fpls.2019.01665)
Supplement: Supplementary file 5 [file DataSheet_5.pdf]

Supplementary Material

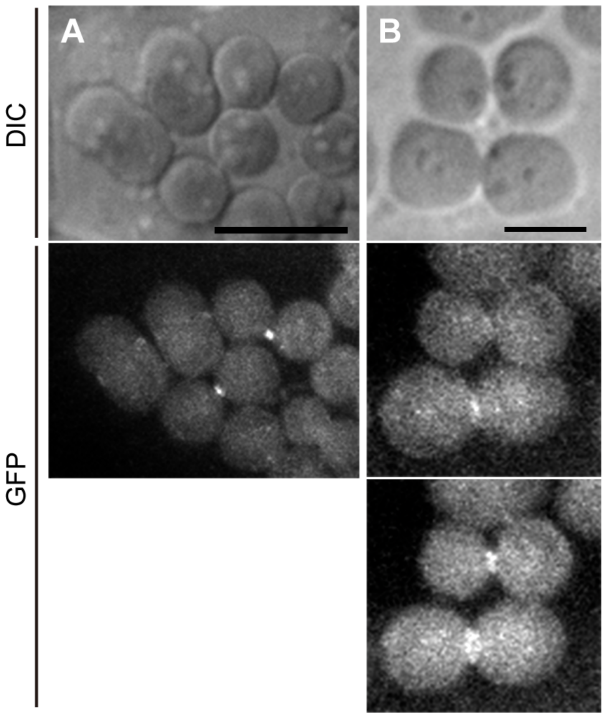

Supplementary Figure S5. Analysis of PARC6-GFP localization in leaf cells. **(A, B)** Images of chloroplasts in leaf petioles of 2-week-old seedlings of *parc6-4* complementation lines. Images of full-length PARC6-GFP and DIC in cortex cells are shown. Scale bar: 10 µm **(A)** and 5 µm (**B**).
